# Risk of suicide in patients with atrial fibrillation receiving different oral anticoagulants: a nationwide analysis using target trial emulation framework

**DOI:** 10.1186/s12916-024-03645-z

**Published:** 2024-10-11

**Authors:** Brian Meng-Hsun Li, Avery Shuei-He Yang, Michael Chun-Yuan Cheng, Huei-Kai Huang, Edward Chia-Cheng Lai

**Affiliations:** 1 School of Pharmacy, Institute of Clinical Pharmacy and Pharmaceutical Sciences, College of Medicine, National Cheng Kung University, Tainan, Taiwan; 2 Population Health Data Center, National Cheng Kung University, Tainan, Taiwan; 3 Department of Family Medicine, Hualien Tzu Chi Hospital, Buddhist Tzu Chi Medical Foundation, Hualien, Taiwan; 4 School of Medicine, Tzu Chi University, Hualien, Taiwan

**Keywords:** Atrial fibrillation, Suicide, Warfarin, NOAC, Vitamin K

## Abstract

**Background:**

The suicide risk in patients with atrial fibrillation receiving novel oral anticoagulants or warfarin has not been evaluated in real-world practice. Moreover, reducing vitamin K levels may increase the suicide risk, underscoring the importance of selecting appropriate oral anticoagulants to prevent unintended outcomes. Therefore, we aimed to evaluate the association between different types of oral anticoagulants and the risk of attempted and completed suicide among patients with atrial fibrillation.

**Methods:**

This nationwide study retrieved data from Taiwan's National Health Insurance Research Database from 2012 to 2020. This study included patients with atrial fibrillation aged 20 years and older who newly received oral anticoagulant treatment, and who had no contraindications for NOACs and no history of suicide-related events. The main outcomes were suicide-related outcomes, including attempted suicide and completed suicide. This study employed the target trial emulation framework to improve the causal inference for the observed association.

**Results:**

A total of 103,768 (71.74%) patients taking NOACs and 40,877 (28.26%) patients taking warfarin were included in this study. Compared to those receiving warfarin, patients receiving NOACs were associated with a lower risk of suicide-related outcomes (HR, 0.82; 95% CIs, 0.69–0.96).

**Conclusions:**

The findings of this cohort study suggested that patients receiving NOACs were associated with a lower risk of suicidal attempts but similar risk of complete suicide, compared to those receiving warfarin. Considering the risk of suicide, NOACs could be the preferred anticoagulants for patients with atrial fibrillation.

**Supplementary Information:**

The online version contains supplementary material available at 10.1186/s12916-024-03645-z.

## Background

Atrial fibrillation (AF) affects a considerable proportion of the global population and is one of the major risk factors associated with the incidence of stroke [1, 2]. Oral anticoagulants are the mainstay in stroke prevention for patients with atrial fibrillation [3, 4]. Warfarin, a vitamin K antagonist, blocks the vitamin K epoxide reductase complex in the liver, thus reducing coagulation. Non-vitamin K antagonist oral anticoagulants (NOACs), which function independently of vitamin K, have been associated with lower risks of major bleeding and fewer drug interactions, compared to warfarin [5].

It has been reported that patients with atrial fibrillation are associated with a higher incidence of psychological symptoms and attempted and completed suicide compared to those with other cardiac conditions, including acute myocardial infarction and angina pectoris [6]. Moreover, animal studies have suggested that reduced vitamin K levels might be linked to increased ceramide levels, leading to psychological symptoms [7, 8]. Clinical studies have also found that the use of warfarin may interfere with vitamin K functions and is associated with a higher risk of anxious and depressive symptoms, compared to NOACs [9–12]. These studies have raised concerns about suicidal tendencies in atrial fibrillation patients, especially in those taking warfarin, because it might interfere with vitamin K functions and negatively affect patients who are already at an increased risk of suicide. Therefore, this study aimed to evaluate the risk of suicide-related outcomes in patients with atrial fibrillation receiving different oral anticoagulants in real-world practice. We hypothesized that, compared to those receiving warfarin, patients receiving NOACs were associated with a lower risk of suicide-related outcomes because NOACs do not interfere with patients’ vitamin K functions. We employed the target trial emulation framework to improve the causal inference for the association observed in this study.

## Methods

### Data source

This nationwide study retrieved data from Taiwan's National Health Insurance Research Database (NHIRD) for the period from 2012 to 2020. The NHIRD includes demographic information and medical claims for outpatient, inpatient and emergency care services of about 23 million people (nearly 99.9% of the entire population of Taiwan) [13, 14]. Several diagnosis codes for major diseases in the claims database have been validated by previous studies, such as ischemic stroke [15], atrial fibrillation [16], and depression [17]. We linked the NHIRD to the Cause of Death Registry which covers the entire population in Taiwan, to capture information about completed suicide. The data for the Cause of Death Register is sourced from government agencies, including the household registration authority and the Ministry of Health and Welfare. This data is based on the cause of death listed on official death certificates, which are legally recognized by the authorized department, ensuring high accuracy of the data. National Cheng Kung University Human Research Ethics Committee approved this study (NCKU HREC-E-110–453-2).

### Specification of the target trial

We hypothesized an open labeled, randomized, pragmatic trial to evaluate the risk of suicide-related outcomes among patients with atrial fibrillation, receiving different oral anticoagulants [18, 19]. The key components of the trial emulation are presented in Additional file 1: Table. S1. Specifically, we included patients with atrial fibrillation aged 20 years and older who newly received an oral anticoagulant, and who had no previous suicide-related events and no contraindications for NOACs. We randomly allocated patients to either NOACs or warfarin. The primary outcomes were suicide-related outcomes, including attempted suicide and completed suicide. The causal contrasts of interest were the intention-to-treat effect and the per-protocol effect [20].

### Emulation of the target trial

We emulated the trial using Taiwan's NHIRD. The study design diagram is provided in Additional file 1: Fig. S1. All eligibility criteria were analog to the target trial. Specifically, the index date was defined as the first date of oral anticoagulant prescription. We retrieved data covering one year before the index date to ensure patients were new users of oral anticoagulants, had no previous suicide-related events, and had no contraindications for NOACs. We confirmed patients’ diagnosis of atrial fibrillation by International Classification of Diseases, Ninth Revision, Clinical Modification codes (ICD-9-CM) codes 427.31 or ICD-10-CM codes I48.0, I48.1, I48.2 and I48.9.

We classified patients into the NOACs or warfarin groups based on their first prescription for oral anticoagulants. To emulate the randomization of the target trial with the two comparison groups sharing the same probability of treatment assignment, we implemented a propensity score with fine stratification weighting approach. The details of this approach are described elsewhere [21, 22]. Briefly, we calculated the propensity score using logistic regression based on the measured covariates listed in Additional file 1: Table S3. These covariates were defined based on literature review and experts’ opinions, including patient age at index date, sex, insurance premium level, index year, specialty of prescriber and hospital level where the oral anticoagulants were prescribed. We also considered patients’ baseline CHA2DS2-VASc score, history of major bleeding events, comorbidities and medication use, which were based on the medical records from one year before the index date (Additional file 1: Fig. S1). After trimming observations from non-overlapping regions of the propensity score distribution, we created 50 equally sized strata based on the PS distribution of the entire population. We then determined the individual patient weights and the average treatment effect among patients receiving NOACs (ATT), according to the numbers of patients in each stratum [21].

### Study outcomes and follow-up period

Just as in the target trial, the primary outcome of the study was suicide-related outcomes, including attempted suicide and completed suicide. Attempted suicide is identified by ICD-9-CM codes E850-E854, E858, E862, E868, E950-E959, 965, 967, 969, 9779 and 986 or ICD-10-CM codes X40, X41, X42, X44, X46, X47, X60-X84, Y87, Y10-Y12, Y16-34, T39, T40, T423, T424, T427, T43, T509 and T58 from both outpatient and inpatient services. These definitions have been used and validated by previous studies [23]. Completed suicide is identified by cause of death Case Type 03 in the Cause of Death Registry. We employed as-started analysis, an analog of intention-to-treat effect, and followed patients from the index date until the occurrence of study outcomes, loss to follow up, death other than suicide or end of the study period (December 31^st^, 2020), whichever occurred first. Also, we employed on-treatment analysis to emulate the per-protocol effect, which additionally censored patients whose treatments changed, including switching to a different anticoagulant and discontinuation (i.e., no prescription refill for more than 90 days from the last day of the previous prescription).

### Statistical analysis

We calculated the standardized mean difference (SMD) to assess differences in the covariates between the two groups. An SMD value of less than 0.1 was considered to signify no difference between the groups [24]. We estimated the cumulative incidence curves for each study outcome using the Kaplan–Meier function. To address possible competing risks from mortality other than suicide, we applied the cause-specific hazard model and the Fine and Gray subdistribution hazard model to compare the risk of suicide-related outcomes between the two groups and we obtained the hazard ratio (HR) and 95% confidence intervals (CIs) for each outcome [25]. All statistical analyses were conducted with SAS software, version 9.4 (SAS Institute).

### Sensitivity analyses

We conducted a series of sensitivity analyses to test the robustness of the analysis. We determined the individual patient weights and the average treatment effect among the entire population (ATE), differing from the targeted population of the main analysis. We classified the NOACs treatment group into four subgroups, i.e., the dabigatran, rivaroxaban, apixaban and edoxaban groups, and evaluated the relative risk for each individual NOAC, compared to warfarin. We repeated the analyses among patients with a history of depressive disorders and among different subgroups by patient age (< 65 or ≥ 65 years), sex (male or female), and index year (2012 to 2014, 2015 to 2017, or 2018 to 2020), to examine if the risk profiles changed [26–28]. Additionally, because clinicians may consider prescribing warfarin for patients with renal impairment, we excluded patients with chronic kidney disease to minimize the potential confounding effect.

We repeated the analyses with positive and negative control outcomes, for which their associations with the drugs were well-known, to examine potential residual confounders. The positive control outcomes included ischemic stroke or systemic embolism, cardiovascular mortality and all-cause mortality [29, 30]. We hypothesized that patients taking NOACs were associated with a reduced risk of these positive control outcomes, compared to warfarin. The negative control outcomes included oral health conditions, which were assumed not to be related to the use of oral anticoagulants. We also performed an analysis of outcomes occurring within 90 days after the index date. We assumed that oral anticoagulants require an induction period of more than 90 days to have an influence on suicide-related outcomes [31]. Any observed difference in the risk of suicide-related outcomes between the groups during this period would likely have resulted from the patients’ baseline risk rather than from the use of oral anticoagulants.

## Results

### Patient characteristics

**Fig. 1:**
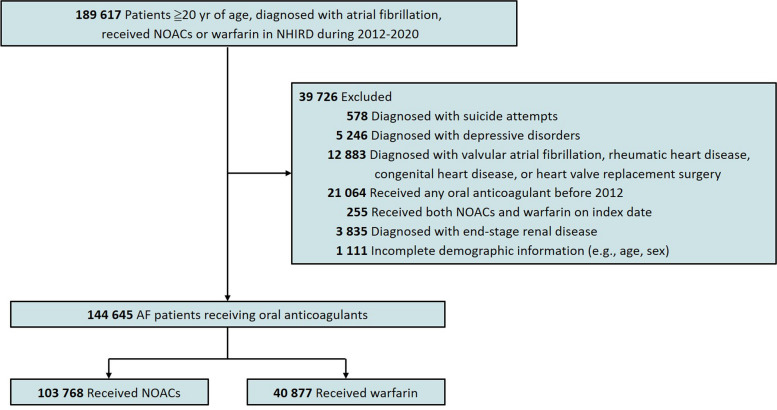
Flow chart of study population selection. NHIRD, national health insurance research database; NOACs, non-vitamin K anticoagulants

We included a total of 103,768 (71.74%) patients initiating NOACs and 40,877 (28.26%) patients initiating warfarin, with a mean age of 74.43 years (SD, 10.97) and 70.58 years (SD, 12.66), respectively (Fig. 1). After applying propensity score methods, the weighted population comprised 103,695 patients in the NOACs group and 40,849 patients in the warfarin group (Additional file 1: Fig. S2). Table 1 shows the baseline characteristics of the weighted cohort with the average treatment effects among the entire population and the treated population. All measured covariates were well balanced between the two treatment groups (Additional file 1: Fig. S3). The overall mean follow‐up durations in the NOACs and warfarin groups were 2.75 and 4.31 years, respectively. The probabilities of discontinuation and switching to different oral anticoagulants were 30.96% and 29.62% in the warfarin group, and 30.83% and 4.28% in the NOACs group, respectively.

**Table 1:** Baseline characteristics of the weighted population

Baseline characteristics^a^	Weighted cohort with ATT			Weighted cohort with ATE		
	**NOACs (*n* = 103,695)**	**Warfarin (*n* = 40,849)**	**SMD**	**NOACs (*n* = 103,695)**	**Warfarin (*n* = 40,849)**	**SMD**
Age, years^b^	74.4 (11.0)	74.5 (12.0)	0.00	73.3 (11.4)	73.4 (12.3)	0.01
Sex						
Male	58706 (56.6)	23167 (56.7)	0.00	59448 (57.3)	23380 (57.2)	0.00
Female	44989 (43.4)	17682 (43.3)	0.00	44247 (42.7)	17469 (42.8)	0.00
Insurance premium level						
< 28,800 NTD	76619 (73.9)	30136 (73.8)	0.00	76126 (73.4)	29961 (73.3)	0.00
28,801–45,800 NTD	13123 (12.7)	5336 (13.1)	0.01	13963 (13.5)	5600 (13.7)	0.01
> 45,800 NTD	13953 (13.5)	5377 (13.2)	0.01	13606 (13.1)	5287 (12.9)	0.01
CHA2DS2-VASc score^b,^^c^	3.8 (1.7)	3.8 (2.0)	0.02	3.7 (1.8)	3.7 (2.0)	0.01
Index year						
2012 to 2014	14174 (13.7)	5693 (13.9)	0.01	26498 (25.6)	10574 (25.9)	0.01
2015 to 2017	40005 (38.6)	15735 (38.5)	0.00	37803 (36.5)	14785 (36.2)	0.01
2018 to 2020	49516 (47.8)	19421 (47.5)	0.00	39394 (38.0)	15489 (37.9)	0.00
Hospital level						
Medical centers	41396 (39.9)	15247 (37.3)	0.05	39436 (38.0)	14865 (36.4)	0.03
Regional hospitals	44630 (43.0)	18785 (46.0)	0.06	45544 (43.9)	18713 (45.8)	0.04
District hospitals or clinics	17669 (17.0)	6816 (16.7)	0.01	18714 (18.0)	7271 (17.8)	0.01
Specialty of prescriber						
Cardiology	74251 (71.6)	29358 (71.9)	0.01	72880 (70.3)	28805 (70.5)	0.01
Neurology	15870 (15.3)	6029 (14.8)	0.02	15518 (15.0)	5926 (14.5)	0.01
Others	13574 (13.1)	5463 (13.4)	0.01	15297 (14.8)	6119 (15)	0.01
Major bleeding events, %	26126 (25.2)	10322 (25.3)	0.00	23794 (22.9)	9453 (23.1)	0.01
Psychiatric comorbidities, %						
Anxiety disorders	10904 (10.5)	4229 (10.4)	0.01	11368 (11.0)	4405 (10.8)	0.01
Bipolar disorders	287 (0.3)	129 (0.3)	0.01	291 (0.3)	126 (0.3)	0.01
Dementia	7497 (7.2)	2813 (6.9)	0.01	7060 (6.8)	2688 (6.6)	0.01
Schizophrenia	463 (0.4)	185 (0.5)	0.00	486 (0.5)	195 (0.5)	0.00
Physical comorbidities						
Anemia	7119 (6.9)	2670 (6.5)	0.01	7059 (6.8)	2701 (6.6)	0.01
Cataract	21126 (20.4)	8332 (20.4)	0.00	20571 (19.8)	8116 (19.9)	0.00
Chronic kidney disease	10940 (10.6)	4194 (10.3)	0.01	11240 (10.8)	4385 (10.7)	0.00
COPD	5593 (5.4)	2391 (5.9)	0.02	8043 (7.8)	3219 (7.9)	0.01
Congestive heart failure	32543 (31.4)	13102 (32.1)	0.02	33990 (32.8)	13559 (33.2)	0.01
Coronary artery disease	37868 (36.5)	14793 (36.2)	0.01	38994 (37.6)	15262 (37.4)	0.01
Deep vein thrombosis	255 (0.2)	121 (0.3)	0.01	371 (0.4)	156 (0.4)	0.00
Diabetes mellitus	32205 (31.1)	12586 (30.8)	0.01	32121 (31.0)	12602 (30.8)	0.00
Epilepsy	1441 (1.4)	548 (1.3)	0.00	1448 (1.4)	553 (1.4)	0.00
Glaucoma	5921 (5.7)	2219 (5.4)	0.01	5615 (5.4)	2137 (5.2)	0.01
Hypertension	64458 (62.2)	24688 (60.4)	0.04	58756 (56.7)	22864 (56.0)	0.01
Hyperthyroidism	3289 (3.2)	1325 (3.2)	0.00	3593 (3.5)	1429 (3.5)	0.00
Hypothyroidism	2135 (2.1)	874 (2.1)	0.02	2134 (2.1)	862 (2.1)	0.00
Hyperlipidemia	36623 (35.3)	14205 (34.8)	0.01	35532 (34.3)	13813 (33.8)	0.01
Malignancy	17135 (16.5)	6552 (16.0)	0.01	16859 (16.3)	6549 (16)	0.01
Osteoporosis	7252 (7.0)	2873 (7.0)	0.00	7014 (6.8)	2760 (6.8)	0.00
Parkinsonism	2938 (2.8)	1161 (2.8)	0.00	2822 (2.7)	1119 (2.7)	0.00
Pulmonary embolism	547 (0.5)	203 (0.5)	0.00	747 (0.7)	305 (0.7)	0.00
Rheumatoid arthritis	1254 (1.2)	463 (1.1)	0.01	1294 (1.2)	478 (1.2)	0.01
Ischemic stroke	21327 (20.6)	8099 (19.8)	0.02	21557 (20.8)	8270 (20.2)	0.01
Medication use						
Antacids	24525 (23.7)	9444 (23.1)	0.01	27435 (26.5)	10669 (26.1)	0.01
Antiarrhythmic drugs	48084 (46.4)	18450 (45.2)	0.02	49469 (47.7)	19187 (47.0)	0.02
Antidepressants	4187 (4.0)	1639 (4.0)	0.00	3920 (3.8)	1541 (3.8)	0.00
Antiepileptics	11915 (11.5)	4796 (11.7)	0.01	11682 (11.3)	4696 (11.5)	0.01
Antiosteoporotic drugs	8796 (8.5)	3301 (8.1)	0.02	9053 (8.7)	3513 (8.6)	0.01
Antiplatelet drugs	70512 (68.0)	28355 (69.4)	0.03	71218 (68.7)	28362 (69.4)	0.02
Antipsychotics	10583 (10.2)	4051 (9.9)	0.01	10500 (10.1)	4074 (10.0)	0.01
Anxiolytics	42952 (41.4)	16853 (41.3)	0.00	43645 (42.1)	17158 (42.0)	0.00
Beta blockers	68960 (66.5)	27109 (66.4)	0.00	69054 (66.6)	27175 (66.5)	0.00
Bronchodilators	15332 (14.8)	6222 (15.2)	0.01	15262 (14.7)	6126 (15.0)	0.01
Calcium-channel blockers	61481 (59.3)	24285 (59.5)	0.00	61838 (59.6)	24446 (59.8)	0.00
Corticosteroids	28992 (28.0)	11418 (28.0)	0.00	29381 (28.3)	11553 (28.3)	0.00
Diuretics	48058 (46.3)	19424 (47.6)	0.02	49575 (47.8)	19876 (48.7)	0.02
H2 blockers	50147 (48.4)	20086 (49.2)	0.02	49554 (47.8)	19744 (48.3)	0.01
Hypnotics and sedatives	21682 (20.9)	8511 (20.8)	0.00	22294 (21.5)	8809 (21.6)	0.00
Hypoglycemic agents	30056 (29.0)	11693 (28.6)	0.01	29978 (28.9)	11746 (28.8)	0.00
Mood stabilizers	1960 (1.9)	763 (1.9)	0.00	1985 (1.9)	781 (1.9)	0.00
NSAIDs	68891 (66.4)	27154 (66.5)	0.00	69487 (67.0)	27400 (67.1)	0.00
Proton pump inhibitors	21634 (20.9)	8482 (20.8)	0.00	21447 (20.7)	8431 (20.6)	0.00
Renin system inhibitors	66437 (64.1)	26152 (64.0)	0.00	65862 (63.5)	25934 (63.5)	0.00
Statins	37787 (36.4)	14652 (35.9)	0.01	35774 (34.5)	13916 (34.1)	0.01

*NOACs* Non-vitamin K antagonist oral anticoagulants, *SMD* Standardized mean differences, *ATT* Average treatment effect among the treated population, *ATE* average treatment effect, *COPD *Chronic obstructive pulmonary disease, *NSAIDs* Non-steroidal anti-inflammatory drugs

Data are expressed as number (%) unless otherwise indicated

^a^All covariates listed in the table were used to calculate the propensity score with fine stratification weighting

^b^Expressed as mean (SD)

^c^Calculated by baseline age [> 65 years = 1 point; > 75 years = 2 points], female, congestive heart failure, hypertension, previous stroke/transient ischemic attack/thromboembolism [2 points], vascular disease, diabetes mellitus

### Suicide-related outcomes

**Fig. 2:**
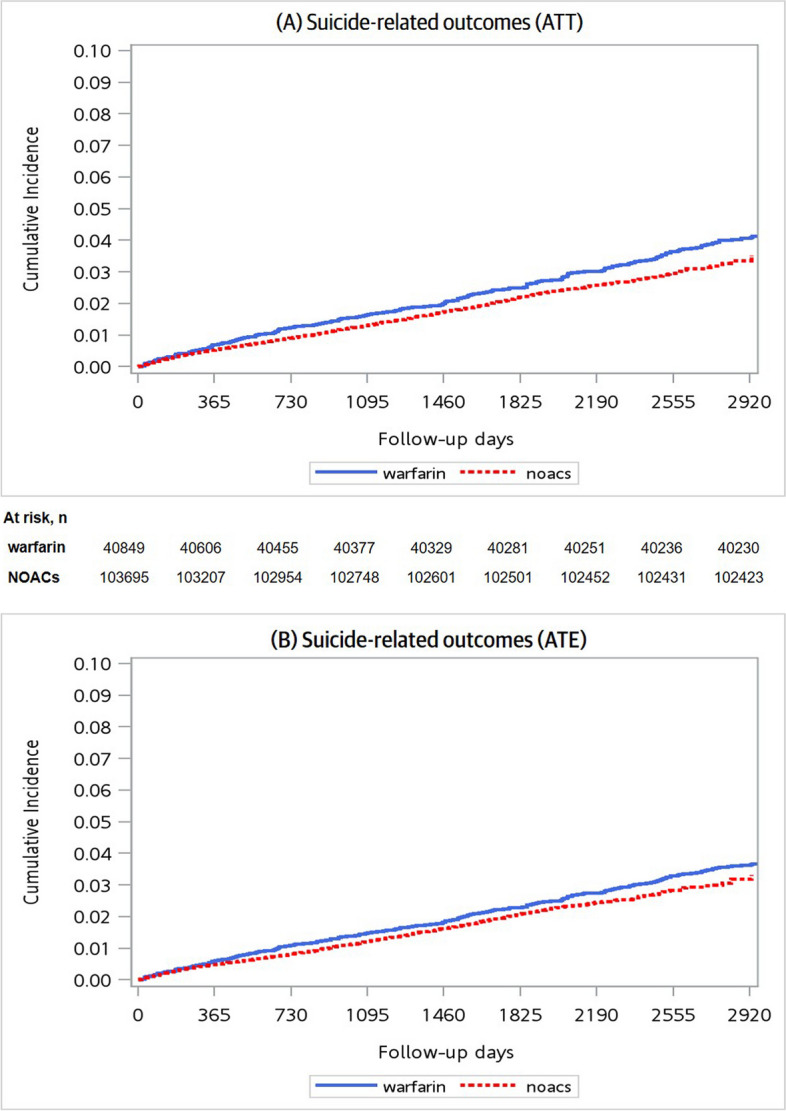
Cumulative incidence curves of suicide-related outcomes in treatment groups. ATT, average treatment effect among the treated population; ATE, average treatment effect. **A** indicates the cumulative incidence curves of suicide-related outcomes in the weighted population, targeting the ATT. **B** indicates the cumulative incidence curves of suicide-related outcomes in the weighted population, targeting the ATE

In the analyses estimating average treatment effect among patients receiving NOACs (ATT), the incidence rates of suicide-related outcomes were 4.49 and 5.50 per 1,000 person-years for NOACs users and warfarin users, respectively (Fig. 2). The HRs (95% CI) for NOACs vs. warfarin were 0.82 (0.69–0.96) for suicide-related outcomes, 0.79 (0.67–0.93) for attempted suicide and 1.27 (0.73–2.20) for completed suicide (Table 2). In the analyses estimating average treatment effect among the entire population (ATE), the incidence rates of suicide-related outcomes were 4.16 and 4.89 per 1,000 person-years for NOACs users and warfarin users, respectively. The HRs (95% CI) for NOACs vs. warfarin were 0.85 (0.75–0.97) for suicide-related outcomes, 0.83 (0.72–0.95) for attempted suicide and 1.17 (0.78–1.76) for completed suicide.

**Table 2:** Risk of suicide-related outcomes in patients treated with NOACs and warfarin

Study outcomes^a^	Weighted cohort with ATT			Weighted cohort with ATE		
	Events, n	Incidence rate^b^	HR (95% CI)	Events, n	Incidence rate^b^	HR (95% CI)
**Primary outcome**						
Suicide-related outcomes						
NOACs	1,274	4.49	0.82 (0.69–0.96)	1,370	4.16	0.85 (0.75–0.97)
warfarin	584	5.50	1.00 (reference)	637	4.89	1.00 (reference)
**Secondary outcomes**						
Attempted suicide						
NOACs	1,157	4.08	0.79 (0.67–0.93)	1,235	3.75	0.83 (0.72–0.95)
warfarin	584	5.81	1.00 (reference)	591	4.54	1.00 (reference)
Completed suicide						
NOACs	128	0.45	1.27 (0.73–2.20)	148	0.45	1.17 (0.78–1.76)
warfarin	40	0.35	1.00 (reference)	49	0.37	1.00 (reference)

*ATT* Average treatment effect among the treated population, *ATE* Average treatment effect

^a^In each outcome analysis, patients who had already experienced the corresponding outcome event before the index date were excluded

^b^Per 1000 person-years

### Sensitivity analyses

**Fig. 3:**
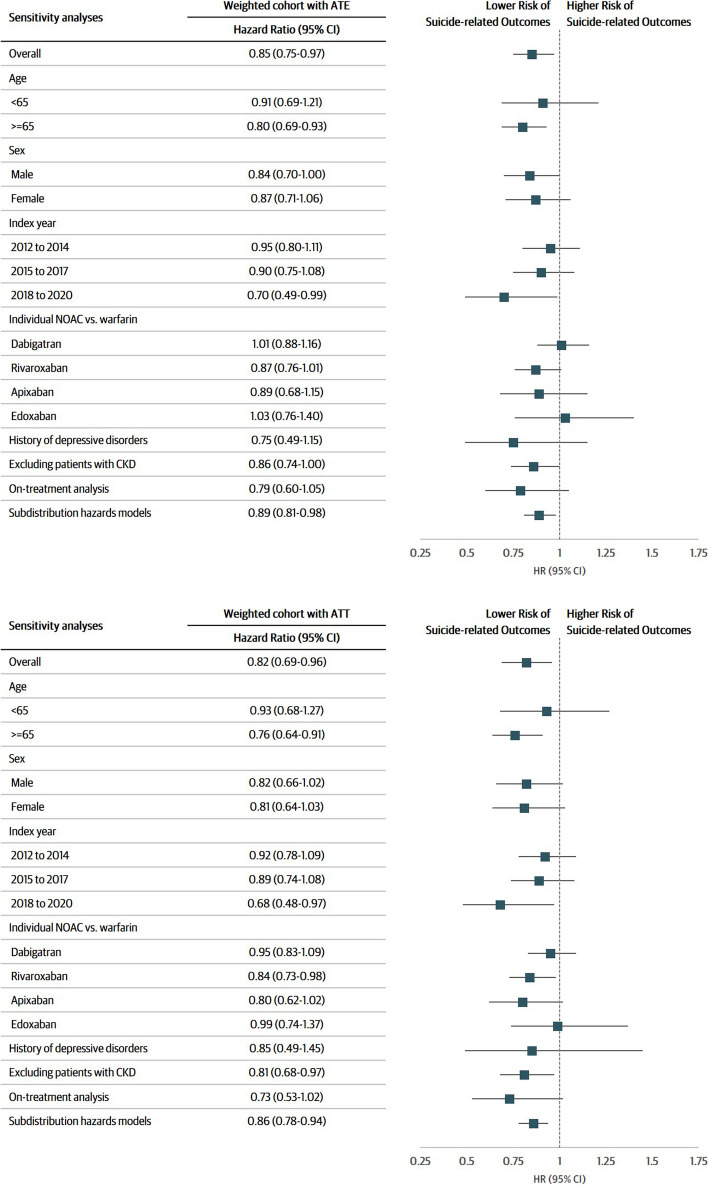
Risk of suicide-related outcomes in sensitivity analyses. ATT, average treatment effect among the treated population; CKD, chronic kidney disease. *Solid line indicates no difference between the two groups (hazard ratio is equal to one). **The *p*-value for interaction terms between age and sex subgroups were 0.2853 and 0.9264, respectively

All sensitivity analyses yielded results consistent with the main analyses (Fig. 3). The analyses for individual NOACs (dabigatran, rivaroxaban and apixaban) showed a lower trend toward suicide-related outcomes in NOACs users, compared to warfarin users. In analyses stratified by different subgroups such as patient age, sex and index year, we observed a lower trend toward suicide-related outcomes in NOACs users, compared to warfarin users. Similarly, among patients with atrial fibrillation and depressive disorders, the results for the risk of suicide-related outcomes remained consistent but less precise (HR: 0.85, 95% CI: 0.49–1.45) (Additional file 1: Table S7-8). After excluding patients with chronic kidney disease, results for the risk of suicide-related outcomes remained consistent with the main analyses (HR: 0.81, 95% CI: 0.68–0.97) (Additional file 1: Table S9-10). Additionally, we conducted on-treatment analyses by censoring patients whose treatments were changed, with a mean follow‐up of 1.67 and 0.91 years for the NOACs and warfarin groups, respectively. The HRs (95% CI) for NOACs vs. warfarin were 0.73 (0.53–1.02) for suicide-related outcomes (Additional file 1: Table S11). The results estimated using the Fine and Gray subdistribution hazard model remained consistent with the main analyses, taking into account possible competing risk from mortality other than suicide (Additional file 1: Table S12).

As regards positive control outcomes, the HRs (95% CI) for NOACs vs. warfarin were 0.81 (0.76–0.86) for ischemic stroke and systemic embolism, 0.76 (0.74–0.79) for all-cause mortality, and 0.70 (0.66–0.73) for cardiovascular mortality (Additional file 1: Table S13). As for negative control outcomes, the HRs (95% CI) for NOACs vs. warfarin were 1.66 (0.86–3.20) for suicide-related outcomes during the first 90 days after initiating oral anticoagulants and 1.00 (0.95–1.05) for oral health-related conditions (Additional file 1: Table S13).

## Discussion

In this nationwide cohort study using target trial emulation framework, the use of NOACs was associated with a lower risk of suicidal attempts but similar risk of complete suicide, compared to the use of warfarin. Furthermore, we obtained consistent results when comparing individual NOACs with warfarin. The results were also robust throughout a series of sensitivity analyses. We did not find any effect modifier in the subgroup analyses by age, sex and other factors in the sensitivity analyses with interaction term. We found the results were also consistent in the analysis of individual NOACs and patients with depressive disorders, although some analyses did not reach statistical significance due to relatively small sample sizes. We considered the results to imply a class effect based on the biological mechanisms of the medication and the etiology of suicide attempts. In summary, these findings suggested that NOACs may be a preferable treatment option for patients with atrial fibrillation, since they are already at an increased risk of suicide.

The association observed in our study between the use of warfarin and an increased risk of suicide could be attributed to both biological etiology and clinical factors. Previous studies have suggested that reduced vitamin K levels may be linked to increased ceramide levels [7]. In vitro experiments have revealed that ceramides can impede the proliferation of neural cells in the hippocampus [32]. Consequently, ceramides inhibit hippocampal neurogenesis and play a crucial role in the negative regulation of the HPA axis and its dysfunction. High levels of ceramides in the brain can lead to unregulated apoptosis of neuronal and oligodendroglial cells, which is associated with psychological symptoms [8]. This biological mechanism may increase the risk of suicide in patients receiving warfarin. Additionally, the use of warfarin has been associated with a higher risk of bleeding, compared to NOACs [29]. This may provide another explanation for the increased risk of suicide-related outcomes, since a higher bleeding risk, along with its need for strict monitoring, can have a negative impact on patients’ quality of life [33–35].

### Implications of the study

Patients with atrial fibrillation are at a higher risk of ischemic stroke and suicide, compared to those without atrial fibrillation [3, 4, 36]. The use of warfarin is associated with an increased risk of psychological symptoms, compared to the use of NOACs [9–12]. These studies have drawn clinical attention to some psychological complications arising during the management of atrial fibrillation and stroke prevention using oral anticoagulants. However, unlike physical symptoms, psychological symptoms are easily neglected in clinical practice, due to insufficient awareness and the absence of routine screening for the detection of psychological symptoms [37]. Our study highlights the importance of clinicians taking into consideration the psychological symptoms of their patients with atrial fibrillation. A routine psychiatric screening using tools such as Patient Health Questionnaire-9 may be beneficial [38, 39]. We have provided evidence that the choice of oral anticoagulants for patients with atrial fibrillation was associated with varying risks of suicide. Based on the present and previous studies, NOACs may be considered a recommended option as they are not only associated with a reduced risk of suicide, but also with decreased risks of ischemic stroke, systemic embolism, bleeding and overall mortality.

### Strengths and limitations

This is the first study using a population-based database to evaluate and compare the risk of suicide-related outcomes among different oral anticoagulants. We adopted the target trial emulation framework to improve the causal inference and the quality of the analysis. We obtained robust results from several sensitivity analyses to support our conclusion. Unlike randomized controlled trials, our retrospective study was able to observe outcomes that required long-term follow-up data. By taking into consideration the induction period of drug effects, our study has bridged a knowledge gap from trials regarding the impact of oral anticoagulant use on long-term suicide-related outcomes.

Our study has several potential limitations. First, misclassification of exposure may have occurred due to drug discontinuation or switching to a different oral anticoagulant, potentially biasing the results towards null. However, the on-treatment analysis, which took drug discontinuation and switching into account, suggested results consistent with the main analysis. Second, our study may have been subject to misclassification of outcomes, especially in cases where patients attempted suicide but sustained only mild injuries that did not require treatment. Although the diagnosis codes have been validated, the sensitivity of the codes may be a concern. However, since the rates of such undocumented suicidal behavior should be nondifferential between the two comparison groups, the potential bias should be towards null. While the risk of suicide may have been underestimated, we still observed a lower risk of suicidal outcomes in NOACs users compared to warfarin users. Additionally, patients receiving warfarin may be more likely to seek healthcare for suicide attempts because evidence has shown that warfarin needs more strict monitoring [33]. We conducted an additional analysis and found no difference in hospital visits between the two groups. The average visits within one year after the index date were 26.33 (SD 17.66) for the NOACs group and 26.60 (SD 18.18) for the warfarin group. Therefore, we considered surveillance bias to be negligible in our study. Third, the results from the analysis restricting the observational period to the first 90 days after initiating oral anticoagulants, indicated that residual confounders cannot be ruled out. As it usually requires an induction period for drugs to produce their effects, any observed difference in suicide risks within the first 90 days is more likely to be related to patients’ underlying conditions. Thus, we may have underestimated the magnitude of suicide risk for those receiving warfarin, given that the baseline suicide risk for patients receiving NOACs appears higher. Fourth, since patients receiving warfarin had relatively high incidences of mortality compared to those receiving NOACs, the potentially competing risk of mortality should be considered. To address this issue, we applied Fine and Gray subdistribution hazard model. Although the Fine and Gray subdistribution hazard model is useful for prediction purposes, the results obtained from using this model imply that the competing risk of mortality cannot explain away our conclusions. Fifth, in observational studies using real-world data, socioeconomic differences can sometimes influence patients' medication choices, thereby affecting study results. However, in our study, we used insurance premium levels as a proxy to adjust for socioeconomic factors, as they are highly correlated with patients' wage levels. Additionally, in Taiwan, financial issues are not expected to affect the choice of anticoagulant type because our national health insurance program fully covers both NOACs and warfarin (including INR testing) with no copayment. Therefore, we believe that socioeconomic status did not affect our results. Finally, a limitation lay in the nature of our study, whereby none of the data was randomized. Although our study considered a large number of covariates and conducted a series of sensitivity analysis to address possible confounders, some residual confounding effect may remain. Moreover, our study predominantly involved a Taiwanese population, and their dosage of warfarin may be lower than that of western populations^40^. Our results’ generalizability to other countries and populations therefore remains uncertain.

## Conclusions

In this nationwide cohort study using target trial emulation framework, the use of NOACs was associated with a lower risk of suicidal attempts but similar risk of complete suicide, compared to the use of warfarin. The results remained consistent across different individual NOACs and through various sensitivity analyses. Given that patients with atrial fibrillation are at an increased risk of suicide, NOACs may present a preferable treatment option over warfarin to minimize the risk of suicide.

## Supplementary Information

Additional file 1: Table S1. Emulation of the target trial. Table S2. Diagnosis codes of study outcomes. Table S3. Definitions of variables. Table S4. Diagnosis codes of indicator variables. Table S5. ATC codes of indicator variables. Table S6. Baseline characteristics of the original population. Table S7. Baseline characteristics among AF patients with depressive disorders. Table S8. Risk of suicide-related outcomes among AF patients with depressive disorders. Table S9. Baseline characteristics excluding AF patients with chronic kidney disease. Table S10. Risk of suicide-related outcomes excluding AF patients with chronic kidney disease. Table S11. Risk of suicide-related outcomes applying on treatment analysis. Table S12. Risk of suicide-related outcomes using subdistribution hazards models. Table S13. Risk of positive control outcomes and negative control outcomes. Fig S1. Study design diagram. Fig S2. Distribution of propensity score. Fig S3. Covariate balance measured by standardized mean differences.

## Data Availability

The authors remotely accessed the data from the data center of the Ministry of Health and Welfare in Taiwan. Researchers interested in accessing this dataset could submit a formal application to the Taiwan Ministry of Health and Welfare to request access (No 488, Sect. 6, Zhongxiao E Rd, Nangang District, Taipei 115, Taiwan; website: https://dep.mohw.gov.tw/DOS/cp-2516-59203-113.html). No additional data available.

## Abbreviations

AF: Atrial fibrillation
ATE: Average treatment effect
ATT: Average treatment effect among the treated population
Cis: Confidence intervals
HR: Hazard ratio
NHIRD: National Health Insurance Research Database
NOACs: Non-vitamin K antagonist oral anticoagulants
SMD: Standardized mean difference

## Data availability

No datasets were generated or analysed during the current study.
