# Supplementary material for: Risk of suicide in patients with atrial fibrillation receiving different oral anticoagulants: a nationwide analysis using target trial emulation framework
Source: BMC Med. 2024 Oct 11;22:451. doi: 10.1186/s12916-024-03645-z (PMC11470551; doi:10.1186/s12916-024-03645-z)
Supplement: Supplementary file 1 — Additional file 1: Table S1. Emulation of the target trial. Table S2. Diagnosis codes of study outcomes. Table S3. Definitions of variables. Table S4. Diagnosis codes of indicator variables. Table S5. ATC codes of indicator variables. Table S6. Baseline characteristics of the original population. Table S7. Baseline characteristics among AF patients with depressive disorders. Table S8. Risk of suicide-related outcomes among AF patients with depressive disorders. Table S9. Baseline characteristics excluding AF patients with chronic kidney disease. Table S10. Risk of suicide-related outcomes excluding AF patients with chronic kidney disease. Table S11. Risk of suicide-related outcomes applying on treatment analysis. Table S12. Risk of suicide-related outcomes using subdistribution hazards models. Table S13. Risk of positive control outcomes and negative control outcomes. Fig S1. Study design diagram. Fig S2. Distribution of propensity score. Fig S3. Covariate balance measured by standardized mean differences. [file 12916_2024_3645_MOESM1_ESM.docx]

### Supplementary Material

Risk of Suicide in Patients with Atrial Fibrillation Receiving Different Oral Anticoagulants: A Nationwide Analysis using Target Trial Emulation Framework

Brian Meng-Hsun Li, MClinPharm; Avery Shuei-He Yang, MClinPharm;
Michael Chun-Yuan Cheng, MS; Huei-Kai Huang, MD; Edward Chia-Cheng Lai1, PhD

**Table S1.** Target trial specification and emulation of the target trial.

**Table S2.** Diagnosis codes of study outcomes.

**Table S3.** Definitions of variables when emulating a target trial.

**Table S4.** Diagnosis codes of indicator variables.

**Table S5.** ATC codes of indicator variables.

**Table S6.** Baseline characteristics of the original population.

**Table S7.** Baseline characteristics of the original population and the weighted population among AF patients with depressive disorders.

**Table S8.** Risk of suicide-related outcomes among AF patients with depressive disorders.

**Table S9.** Baseline characteristics of the original population and the weighted population excluding AF patients with chronic kidney disease.

**Table S10.** Risk of suicide-related outcomes excluding AF patients with chronic kidney disease.

**Table S11.** Risk of suicide-related outcomes applying on treatment analysis.

**Table S12.** Risk of suicide-related outcomes using subdistribution hazards models.

**Table S13.** Risk of positive control outcomes and negative control outcomes.

**Fig S1.** Study design diagram.

**Fig S2.** Distribution of propensity score before and after propensity score weighting.

**Fig S3.** Covariate balance measured by standardized mean differences before and after propensity score weighting.

This supplemental material has been provided by the authors to give readers additional information about their work.

| **Table S1.** Target trial specification and emulation of the target trial. | | |
| --- | --- | --- |
| **Protocol**  **component** | **Target trial specification** | **Emulation of the target trial** |
| Aim | To evaluate new use of NOACs and warfarin for suicide-related outcomes among patients with AF | Same as the target trial |
| Eligibility  criteria | - Aged ≥20 years between January 1, 2012, and December 31, 2020 - Previously diagnosed with AF - No previous diagnosis of attempted suicides - No previous diagnosis of depressive disorder - No previous prescription of OACs - No contraindication for NOACs   - Previous diagnosis of valvular atrial fibrillation   - Previous diagnosis of end-stage renal disease | Same as the target trial, except:   - We excluded patients with previous diagnosis of valvular atrial fibrillation, rheumatic heart disease, congenital heart disease, or who had received heart valve replacement surgery because these diagnoses are related to valvular atrial fibrillation. |
| Treatment strategies | 1. Initially receiving NOACs, or 2. Initially receiving warfarin | Same as the target trial |
| Treatment assignment | Eligible patients are randomly assigned to either treatment group with open-label approach. (the same probability of treatment assignment between the two groups) | Using propensity score with fine stratification weighting to generate a study population with balance characteristics (the similar probability of treatment assignment between the two groups) |
| Outcomes | - Suicide-related outcomes (including attempted suicide and successful suicide) - Attempted suicide - Successful suicide | Same as the target trial |
| Follow-up | For each person, follow-up begins at treatment assignment and ends at occurrence of study outcomes, at loss to follow up, at death, or the end of the study period (December 31, 2020), whichever occurs first. | Same as the target trial |
| Causal  contrasts | Main analysis: ITT effect  (i.e., effect of being assigned to NOAC versus warfarin at baseline, regardless of whether patients continue following the assigned treatment after baseline) | Same (using as-started analysis, analog of ITT) |
|  | Sensitivity analysis: Per-protocol effect  (i.e., effect of following the treatment strategies in the study protocol at baseline and after baseline) | Same  (using on-treatment analysis, analog of per-protocol)   - Follow-up begins at treatment assignment and ends at occurrence of treatment changes, study outcomes, at death, or on 31 December 2020, whichever occurs first. - Treatment changes are defined as   - Discontinuation (no prescription refill for more than 90 days from the last day of the previous prescription)   - Switching (patients with the different group of oral anticoagulant prescription) |
| Statistical  analysis | Cox proportional hazards models to generate hazard ratios for each outcome with 95% confidence intervals. (time-to-event risk) | Same as the target trial |

| **Table S2.** Diagnosis codes of study outcomes. | | |
| --- | --- | --- |
| **Variable** | **ICD-9 CM codes** | **ICD-10 CM codes** |
| Self-inflicted poisoning | E950-E952 | X60-X69 |
| Self-inflicted injury | E953-E958 | X70-X84 |
| Late effects of self-inflicted injury | E959 | Y87 |
| Accidental poisoning | E850-E854, E858, E862, E868 | X40, X41, X42, X44, X46, X47 |
| Poisoning of undetermined intent |  | Y10-Y12, Y16-19 |
| Other events of undetermined intent |  | Y20-Y34 |
| Poisoning by drugs, medicaments and biological substances | 965, 967, 969, 9779, 986 | T39, T40, T423, T424, T427, T43, T509, T58 |
| Successful suicide* | Case type: 3 | Case type: 3 |
| Ishemic stroke or systemic embolism | 433x1, 434x1, 436, 444, 445 | I63, I64, I74 |
| Cardiovascular mortality† | 390–459 | I00–I99 |
| Oral health-related conditions | 520-523, 525, 527-529 | K00-K14 |

* Using the manner of death (case type 3 as successful suicide) in National Register of Death.

† Using the primary diagnosis for the cause of death in National Register of Death.

| **Table S3.** Definitions of measured covariates when emulating a target trial. | | |
| --- | --- | --- |
| **Covariate** | **Functional form** | **Values** |
| Age | Linear | N/A |
| Sex | Indicator | Male/Female |
| Insurance premium level | 3 categories | <28,800,  28,801 to 45,800,  >45,800 |
| CHA2DS2-VASc score | Linear | N/A  (calculated using the demographics and comorbidities at the baseline) |
| Index year | 3 categories | 2012 to 2014,  2015 to 2017,  2018 to 2020 |
| Hospital level | 3 categories | Medical center,  Regional center,  District hospital or clinics |
| Specialty of prescriber | 3 categories | Cardiology,  Neurology,  Others |
| Major bleeding events | Indicator | Yes/No (defined as ≥1 inpatient or outpatient diagnosis within the year before index date) |
| Psychiatric/ physical comorbidities | Indicator | Yes/No (defined as ≥1 inpatient or outpatient diagnosis within the year before index date) |
| Medication use | Indicator | Yes/No (defined as ≥1 inpatient or outpatient diagnosis within the year before index date) |

| **Table S4.** Diagnosis codes of indicator variables. | | |
| --- | --- | --- |
| **Variable** | **ICD-9 CM codes** | **ICD-10 CM codes** |
| Major bleeding events | 430, 431, 4320, 4321, 4329, 5307, 531, 5312, 5314, 5316, 532, 5322, 5324, 5326, 533, 5332, 5334, 5336, 534, 5342, 5344, 5346, 5693, 53501, 53511, 53521, 53531, 53541, 53551, 53561, 53571, 53771, 53783, 53784, 56202, 56203, 56212, 56213, 56985, 578, 3361, 3636, 37272, 37632, 37742, 37923, 59381, 86601, 86602, 86611, 86612, 7191, 72992, 4230, 7725 | I60, I61, I62, K226, K25-K28, K2901, K2921, K2931, K2941, K2951, K2961, K2971, K2981, K2991, K31811, K3182, K5281, K5521, K5660, K5701, K5711, K5713, K5721, K5731, K5733, K5781, K5791, K5793, K625, K920, K921, K922, G9511, G9519, H0523, H113, H313, H431, H4702, I312, M250, N280, P544, S31001A, S37011A, S37012A, S37019A, S37021A, S37022A, S37029A, S37031A, S37032A, S37039A, S37041A, S37042A, S37049A, S37051A, S37052A, S37059A |
| Alcohol and tobacco use disorders | 2910, 29181-29189, 2919, 3030-30393, 30500-30503 | F10, F17 |
| Anxiety disorders | 29384, 3000, 3002-3003, 3009, 3083, 30921, 30981, 3130, 31321, 31323 | F40, F41 |
| Bipolar disorders | 2960-2961, 2964-2967, 29680-81 | F30, F31 |
| Dementia | 2900-2909, 2941, 2942, 2948, 3310-3311, 33182 | F00, F01, F01, F02, F03 |
| Schizophrenia | 2950-2959, 297, 2981, 2983-2989 | F20, F22, F23, F25, F28, F29 |
| Anemia | 2801, 2808, 2809, 281, 28401, 28409, 2848, 2849, 2859 | D50-D53, D55-D59, D60-D64 |
| Cataract | 366 | H25-H26, H28 |
| Chronic kidney disease | 585 | N18 |
| Chronic obstructive pulmonary disease | 491, 492, 496 | J41-J44 |
| Congestive heart failure | 428 | I50 |
| Coronary artery disease | 410-414 | I20-I25 |
| Deep vein thrombosis | 4511, 4512, 45181, 45183 | I801, I802, I803 |
| Diabetes mellitus | 2500-2509 | E10-E14 |
| Epilepsy | 3450-3459 | G40-G41 |
| Hyperthyroidism | 242 | E05 |
| Hypothyroidism | 244 | E018, E02, E03, E890 |
| Hypertension | 402-405 | I10-I15 |
| Hyperlipidemia | 272 | E78 |
| Malignancy | 140-239 | C00-C97, D00-D48 |
| Osteoporosis | 733 | M80-M82 |
| Parkinsonism | 3320-3321 | G20, G21 |
| Pulmonary embolism | 4151 | I260, I269 |
| Rheumatoid arthritis | 7140-7149 | M05, M06 |
| Ischemic Stroke | 433x1, 434x1, 436 | I63, I64 |

| **Table S5.** ATC codes of indicator variables. | |
| --- | --- |
| **Variable** | **ATC codes** |
| Antacids | A02AG, A02BX13, A02AX |
| Antiarrhythmic drugs | C01BA, C01BB, C01BC, C01BD01, C01BD07 |
| Antidepressants | N06AB03, N06AB04, N06AB05, N06AB06, N06AB08, N06AB10, N06AX16, N06AX17, N06AX21, N06AX11, N06AX12, N06AX22, N06AX26, N06AG02 |
| Antiepileptics | N03 (exclude N03AG01, N03AF01, N03AX09) |
| Antiosteoporotic drugs | M05B, H05AA, H05BA, A11CC, A12A, G03C, G03F, G03X |
| Antiplatelet drugs | B01AC04, B01AC05, B01AC06, B01AC07, B01AC22, B01AC23, B01AC24, B01AC30 |
| Antipsychotics | N05A (exclude N05AN, N05AB04) |
| Anxiolytics | N05BA |
| Beta blockers | C07 |
| Bronchodilators | R03AL, R03AK |
| Calcium-channel blockers | C08 |
| Corticosteroids | D07 |
| Diuretics | C03 |
| H2 blockers | A02BA |
| Hypnotics and sedatives | N05CD, N05CF |
| Hypoglycemic agents | A10 |
| Mood stabilizers | N05AN01, N03AG01, N03AF01, N03AX09 |
| NSAIDs | M01AB, M01AC, M01AE, M01AH, M01AX |
| Proton pump inhibitors | A02BC |
| Renin system inhibitors | C09 |
| Statins | C10AA |

| **Table S6.** Baseline characteristics of the original population. | | | |
| --- | --- | --- | --- |
| **Baseline characteristics*** | **Original cohort** | | |
|  | **NOACs**  **(n=103,695)** | **Warfarin**  **(n=40,849)** | **SMD** |
| Age, years† | 74.4 (11.0) | 70.6 (12.7) | 0.33 |
| Sex |  |  |  |
| Male | 58754 (56.6) | 23940 (58.6) | 0.04 |
| Female | 45014 (43.4) | 16937 (41.4) | 0.04 |
| Insurance premium level |  |  |  |
| <28,800 NTD | 76690 (73.9) | 29535 (72.3) | 0.04 |
| 28,801-45,800 NTD | 13123 (12.6) | 6278 (15.4) | 0.08 |
| >45,800 NTD | 13955 (13.4) | 5064 (12.4) | 0.03 |
| CHA2DS2-VASc score†§ | 3.8 (1.7) | 3.4 (2.0) | 0.23 |
| Index year |  |  |  |
| 2012 to 2014 | 14174 (13.7) | 22993 (56.2) | 1.00 |
| 2015 to 2017 | 40005 (38.6) | 12375 (30.3) | 0.18 |
| 2018 to 2020 | 49589 (47.8) | 5509 (13.5) | 0.80 |
| Hospital level |  |  |  |
| Medical centers | 41464 (40.0) | 13897 (34.0) | 0.12 |
| Regional hospitals | 44635 (43.0) | 18542 (45.4) | 0.05 |
| District hospitals or clinics | 17669 (17.0) | 8438 (20.6) | 0.09 |
| Specialty of prescriber |  |  |  |
| Cardiology | 74306 (71.6) | 27407 (67.0) | 0.10 |
| Neurology | 15888 (15.3) | 5664 (13.9) | 0.04 |
| Others | 13574 (13.1) | 7806 (19.1) | 0.16 |
| Major bleeding events | 26158 (25.2) | 7252 (17.7) | 0.18 |
| Psychiatric comorbidities |  |  |  |
| Anxiety disorders | 10905 (10.5) | 4856 (11.9) | 0.04 |
| Bipolar disorders | 287 (0.3) | 117 (0.3) | 0.00 |
| Dementia | 7509 (7.2) | 2374 (5.8) | 0.06 |
| Schizophrenia | 463 (0.4) | 225 (0.6) | 0.02 |
| Physical comorbidities |  |  |  |
| Anemia | 7119 (6.9) | 2780 (6.8) | 0.00 |
| Cataract | 21151 (20.4) | 7570 (18.5) | 0.05 |
| Chronic kidney disease | 10940 (10.5) | 4879 (11.9) | 0.04 |
| COPD | 5593 (5.4) | 5326 (13.0) | 0.27 |
| Congestive heart failure | 32558 (31.4) | 14733 (36.0) | 0.10 |
| Coronary artery disease | 37885 (36.5) | 16466 (40.3) | 0.08 |
| Deep vein thrombosis | 255 (0.2) | 251 (0.6) | 0.06 |
| Diabetes mellitus | 32219 (31.0) | 12646 (30.9) | 0.00 |
| Epilepsy | 1445 (1.4) | 567 (1.4) | 0.00 |
| Glaucoma | 5930 (5.7) | 1928 (4.7) | 0.05 |
| Hypertension | 64521 (62.2) | 18240 (44.6) | 0.36 |
| Hyperthyroidism | 3289 (3.2) | 1701 (4.2) | 0.05 |
| Hypothyroidism | 2137 (2.1) | 832 (2.0) | 0.00 |
| Hyperlipidemia | 36658 (35.3) | 12820 (31.4) | 0.08 |
| Malignancy | 17149 (16.5) | 6545 (16.0) | 0.01 |
| Osteoporosis | 7269 (7.0) | 2475 (6.1) | 0.04 |
| Parkinsonism | 2945 (2.8) | 1014 (2.5) | 0.02 |
| Pulmonary embolism | 547 (0.5) | 575 (1.4) | 0.09 |
| Rheumatoid arthritis | 1254 (1.2) | 516 (1.3) | 0.01 |
| Ischemic stroke | 21346 (20.6) | 8706 (21.3) | 0.02 |
| Medication use |  |  |  |
| Antacids | 24528 (23.6) | 13793 (33.7) | 0.23 |
| Antiarrthymic drugs | 48088 (46.3) | 21076 (51.6) | 0.11 |
| Antidepressants | 4196 (4.0) | 1291 (3.2) | 0.05 |
| Antiepileptics | 11934 (11.5) | 4445 (10.9) | 0.02 |
| Antiosteoporotic drugs | 8800 (8.5) | 4057 (9.9) | 0.05 |
| Antiplatelet drugs | 70578 (68.0) | 28391 (69.5) | 0.03 |
| Antipsychotics | 10588 (10.2) | 4138 (10.1) | 0.00 |
| Anxiolytics | 42975 (41.4) | 17948 (43.9) | 0.05 |
| Beta blockers | 68999 (66.5) | 27359 (66.9) | 0.01 |
| Bronchodilators | 15352 (14.8) | 5882 (14.4) | 0.01 |
| Calcium-channel blockers | 61531 (59.3) | 24873 (60.8) | 0.03 |
| Corticosteroids | 29017 (28.0) | 11905 (29.1) | 0.03 |
| Diuretics | 48086 (46.3) | 21037 (51.5) | 0.10 |
| H2 blockers | 50186 (48.4) | 18887 (46.2) | 0.04 |
| Hypnotics and sedatives | 21693 (20.9) | 9573 (23.4) | 0.06 |
| Hypoglycemic agents | 30070 (29.0) | 11884 (29.1) | 0.00 |
| Mood stabilizers | 1963 (1.9) | 828 (2.0) | 0.01 |
| NSAIDs | 68940 (66.4) | 28044 (68.6) | 0.05 |
| Proton pump inhibitors | 21647 (20.9) | 8309 (20.3) | 0.01 |
| Renin system inhibitors | 66504 (64.1) | 25390 (62.1) | 0.04 |
| Statins | 37833 (36.5) | 12050 (29.5) | 0.15 |

NOACs, non-vitamin K antagonist oral anticoagulants; SMD, standardized mean differences; ATE, average treatment effect; ATT, average treatment effect among the treated population; COPD, chronic obstructive pulmonary disease; NSAIDs, non-steroidal anti-inflammatory drugs.

* All the covariates listed in the table were used to calculate the propensity score with fine stratification weighting.

Data are expressed as number (%) unless otherwise indicated.

† Expressed as mean (SD)

§ Calculated by baseline age [>65 years = 1 point; >75 years =2 points], female, congestive heart failure, hypertension, previous stroke/transient ischemic attack/thromboembolism [2 points], vascular disease, diabetes mellitus

| **Table S7.** Baseline characteristics of the original population and the weighted population among AF patients with depressive disorders. | | | | | | | | | |
| --- | --- | --- | --- | --- | --- | --- | --- | --- | --- |
| **Baseline characteristics*** | **Original cohort** | | | **Weighted cohort with ATE** | | | **Weighted cohort with ATT** | | |
|  | **NOACs**  **(n=3,507)** | **Warfarin**  **(n=1,739)** | **SMD** | **NOACs**  **(n=3,484)** | **Warfarin**  **(n=1,730)** | **SMD** | **NOACs**  **(n=3,484)** | **Warfarin**  **(n=1,730)** | **SMD** |
| Age, years† | 76 (10.2) | 72.7 (11.3) | 0.31 | 76 (10.1) | 75.7 (11.4) | 0.03 | 74.8 (10.7) | 74.7 (11.5) | 0.01 |
| Sex |  |  |  |  |  |  |  |  |  |
| Male | 1539 (43.9) | 763 (43.9) | 0.00 | 1522 (43.7) | 831 (48.0) | 0.09 | 1530 (43.9) | 808 (46.7) | 0.06 |
| Female | 1968 (56.1) | 976 (56.1) | 0.00 | 1962 (56.3) | 899 (52.0) | 0.09 | 1954 (56.1) | 922 (53.3) | 0.06 |
| Insurance premium level |  |  |  |  |  |  |  |  |  |
| <28,800 NTD | 2700 (77.0) | 1270 (73.0) | 0.09 | 2681 (77.0) | 1326 (76.6) | 0.01 | 2657 (76.3) | 1306 (75.5) | 0.02 |
| 28,801-45,800 NTD | 376 (10.7) | 254 (14.6) | 0.12 | 374 (10.7) | 176 (10.2) | 0.02 | 407 (11.7) | 201 (11.6) | 0.00 |
| >45,800 NTD | 431 (12.3) | 215 (12.4) | 0.00 | 429 (12.3) | 228 (13.2) | 0.03 | 419 (12.0) | 223 (12.9) | 0.03 |
| CHA2DS2-VASc score†§ | 4.2 (1.7) | 3.9 (2.0) | 0.16 | 4.2 (1.7) | 4.2 (2.0) | 0.01 | 4.1 (1.7) | 4.1 (2.0) | 0.01 |
| Index year, % |  |  |  |  |  |  |  |  |  |
| 2012 to 2014 | 709 (20.2) | 1129 (64.9) | 1.01 | 708 (20.3) | 352 (20.3) | 0.00 | 1230 (35.3) | 607 (35.1) | 0.01 |
| 2015 to 2017 | 1541 (43.9) | 480 (27.6) | 0.35 | 1541 (44.2) | 774 (44.7) | 0.01 | 1344 (38.6) | 677 (39.1) | 0.01 |
| 2018 to 2020 | 1257 (35.8) | 130 (7.5) | 0.73 | 1235 (35.4) | 604 (34.9) | 0.01 | 910 (26.1) | 447 (25.8) | 0.01 |
| Hospital level |  |  |  |  |  |  |  |  |  |
| Medical centers | 1431 (40.8) | 623 (35.8) | 0.10 | 1417 (40.7) | 586 (33.9) | 0.14 | 1354 (38.9) | 599 (34.6) | 0.09 |
| Regional hospitals | 1542 (44) | 751 (43.2) | 0.03 | 1533 (44.0) | 849 (49.1) | 0.10 | 1566 (45.0) | 814 (47.1) | 0.04 |
| District hospitals or clinics | 534 (15.2) | 365 (21) | 0.15 | 534 (15.3) | 295 (17.0) | 0.05 | 564 (16.2) | 317 (18.3) | 0.06 |
| Specialty of prescriber |  |  |  |  |  |  |  |  |  |
| Cardiology | 2396 (68.3) | 1084 (62.3) | 0.13 | 2380 (68.3) | 1184 (68.4) | 0.00 | 2308 (66.3) | 1150 (66.5) | 0.01 |
| Neurology | 621 (17.7) | 241 (13.9) | 0.11 | 615 (17.7) | 281 (16.3) | 0.04 | 573 (16.4) | 268 (15.5) | 0.03 |
| Others | 490 (14.0) | 414 (23.8) | 0.25 | 489 (14.0) | 265 (15.3) | 0.04 | 603 (17.3) | 312 (18) | 0.02 |
| Major bleeding events | 1087 (31.0) | 367 (21.1) | 0.23 | 1074 (30.8) | 547 (31.6) | 0.02 | 951 (27.3) | 487 (28.2) | 0.02 |
| Psychiatric comorbidities |  |  |  |  |  |  |  |  |  |
| Anxiety disorders | 1423 (40.6) | 734 (42.2) | 0.03 | 1415 (40.6) | 745 (43.1) | 0.05 | 1436 (41.2) | 740 (42.8) | 0.03 |
| Bipolar disorders | 101 (2.9) | 44 (2.5) | 0.02 | 99 (2.8) | 56 (3.2) | 0.02 | 91 (2.6) | 52 (3.0) | 0.02 |
| Dementia | 842 (24.0) | 376 (21.6) | 0.06 | 837 (24.0) | 424 (24.5) | 0.01 | 810 (23.2) | 408 (23.6) | 0.01 |
| Schizophrenia | 127 (3.6) | 52 (3.0) | 0.04 | 126 (3.6) | 59 (3.4) | 0.01 | 129 (3.7) | 57 (3.3) | 0.02 |
| Physical comorbidities |  |  |  |  |  |  |  |  |  |
| Anemia | 338 (9.6) | 179 (10.3) | 0.02 | 338 (9.7) | 147 (8.5) | 0.04 | 345 (9.9) | 158 (9.1) | 0.03 |
| Cataract | 900 (25.7) | 440 (25.3) | 0.01 | 894 (25.7) | 438 (25.3) | 0.01 | 889 (25.5) | 439 (25.4) | 0.00 |
| Chronic kidney disease | 424 (12.1) | 257 (14.8) | 0.08 | 423 (12.1) | 214 (12.4) | 0.01 | 448 (12.9) | 227 (13.1) | 0.01 |
| COPD | 357 (10.2) | 301 (17.3) | 0.21 | 357 (10.2) | 205 (11.8) | 0.05 | 456 (13.1) | 236 (13.6) | 0.02 |
| Congestive heart failure | 1070 (30.5) | 599 (34.4) | 0.08 | 1062 (30.5) | 546 (31.5) | 0.02 | 1126 (32.3) | 562 (32.5) | 0.00 |
| Coronary artery disease | 1461 (41.7) | 817 (47) | 0.11 | 1454 (41.7) | 751 (43.4) | 0.03 | 1525 (43.8) | 771 (44.6) | 0.02 |
| Deep vein thrombosis | 14 (0.4) | 21 (1.2) | 0.09 | 14 (0.4) | 6 (0.3) | 0.01 | 25 (0.7) | 11 (0.6) | 0.01 |
| Diabetes mellitus | 1129 (32.2) | 576 (33.1) | 0.02 | 1124 (32.3) | 579 (33.5) | 0.03 | 1146 (32.9) | 577 (33.4) | 0.01 |
| Epilepsy | 77 (2.2) | 53 (3.0) | 0.05 | 77 (2.2) | 34 (2.0) | 0.02 | 87 (2.5) | 41 (2.3) | 0.01 |
| Glaucoma | 258 (7.4) | 117 (6.7) | 0.03 | 257 (7.4) | 113 (6.5) | 0.03 | 246 (7.1) | 114 (6.6) | 0.02 |
| Hypertension | 2134 (60.8) | 826 (47.5) | 0.27 | 2116 (60.7) | 1047 (60.5) | 0.01 | 1958 (56.2) | 973 (56.2) | 0.00 |
| Hyperthyroidism | 131 (3.7) | 85 (4.9) | 0.06 | 131 (3.8) | 50 (2.9) | 0.05 | 144 (4.1) | 62 (3.6) | 0.03 |
| Hypothyroidism | 142 (4) | 65 (3.7) | 0.02 | 139 (4) | 66 (3.8) | 0.01 | 134 (3.9) | 65 (3.8) | 0.01 |
| Hyperlipidemia | 1299 (37.0) | 608 (35.0) | 0.04 | 1290 (37) | 667 (38.6) | 0.03 | 1270 (36.4) | 647 (37.4) | 0.02 |
| Malignancy | 682 (19.4) | 341 (19.6) | 0.00 | 680 (19.5) | 309 (17.9) | 0.04 | 665 (19.1) | 318 (18.4) | 0.02 |
| Osteoporosis | 422 (12.0) | 195 (11.2) | 0.03 | 419 (12) | 199 (11.5) | 0.02 | 410 (11.8) | 197 (11.4) | 0.01 |
| Parkinsonism | 317 (9.0) | 122 (7.0) | 0.08 | 313 (9) | 141 (8.1) | 0.03 | 294 (8.4) | 134 (7.7) | 0.03 |
| Pulmonary embolism | 19 (0.5) | 38 (2.2) | 0.14 | 19 (0.5) | 8 (0.4) | 0.02 | 36 (1.0) | 16 (0.9) | 0.01 |
| Rheumatoid arthritis | 56 (1.6) | 43 (2.5) | 0.06 | 56 (1.6) | 37 (2.1) | 0.04 | 63 (1.8) | 39 (2.2) | 0.03 |
| Ischemic stroke | 855 (24.4) | 462 (26.6) | 0.05 | 848 (24.3) | 401 (23.2) | 0.03 | 901 (25.9) | 420 (24.3) | 0.04 |
| Medication use |  |  |  |  |  |  |  |  |  |
| Antacids | 1072 (30.6) | 716 (41.2) | 0.22 | 1069 (30.7) | 543 (31.4) | 0.02 | 1189 (34.1) | 600 (34.7) | 0.01 |
| Antiarrthymic drugs | 1772 (50.5) | 996 (57.3) | 0.14 | 1769 (50.8) | 843 (48.7) | 0.04 | 1824 (52.4) | 892 (51.5) | 0.02 |
| Antidepressants | 2471 (70.5) | 1191 (68.5) | 0.04 | 2454 (70.4) | 1266 (73.2) | 0.06 | 2427 (69.6) | 1238 (71.6) | 0.04 |
| Antiepileptics | 1138 (32.4) | 597 (34.3) | 0.04 | 1134 (32.5) | 565 (32.7) | 0.00 | 1173 (33.7) | 574 (33.2) | 0.01 |
| Antiosteoporotic drugs | 513 (14.6) | 277 (15.9) | 0.04 | 510 (14.6) | 219 (12.7) | 0.06 | 513 (14.7) | 238 (13.7) | 0.03 |
| Antiplatelet drugs | 2528 (72.1) | 1314 (75.6) | 0.08 | 2506 (71.9) | 1320 (76.3) | 0.10 | 2554 (73.3) | 1316 (76.1) | 0.06 |
| Antipsychotics | 1314 (37.5) | 612 (35.2) | 0.05 | 1303 (37.4) | 664 (38.4) | 0.02 | 1273 (36.5) | 646 (37.3) | 0.02 |
| Anxiolytics | 2641 (75.3) | 1377 (79.2) | 0.09 | 2628 (75.4) | 1325 (76.6) | 0.03 | 2668 (76.6) | 1340 (77.4) | 0.02 |
| Beta blockers | 2468 (70.4) | 1295 (74.5) | 0.09 | 2462 (70.7) | 1284 (74.2) | 0.08 | 2508 (72.0) | 1286 (74.3) | 0.05 |
| Bronchodilators | 610 (17.4) | 287 (16.5) | 0.02 | 605 (17.4) | 338 (19.5) | 0.06 | 609 (17.5) | 321 (18.5) | 0.03 |
| Calcium-channel blockers | 2334 (66.6) | 1191 (68.5) | 0.04 | 2316 (66.5) | 1157 (66.9) | 0.01 | 2349 (67.4) | 1167 (67.4) | 0.00 |
| Corticosteroids | 1275 (36.4) | 658 (37.8) | 0.03 | 1270 (36.5) | 646 (37.3) | 0.02 | 1282 (36.8) | 649 (37.5) | 0.02 |
| Diuretics | 1716 (48.9) | 934 (53.7) | 0.10 | 1705 (48.9) | 865 (50.0) | 0.02 | 1783 (51.2) | 886 (51.2) | 0.00 |
| H2 blockers | 2072 (59.1) | 973 (56.0) | 0.06 | 2058 (59.1) | 1110 (64.2) | 0.11 | 2032 (58.3) | 1063 (61.5) | 0.06 |
| Hypnotics and sedatives | 2155 (61.4) | 1120 (64.4) | 0.06 | 2142 (61.5) | 1120 (64.7) | 0.07 | 2192 (62.9) | 1118 (64.6) | 0.04 |
| Hypoglycemic agents | 995 (28.4) | 527 (30.3) | 0.04 | 990 (28.4) | 508 (29.3) | 0.02 | 1007 (28.9) | 513 (29.6) | 0.02 |
| Mood stabilizers | 179 (5.1) | 114 (6.6) | 0.06 | 178 (5.1) | 89 (5.1) | 0.00 | 180 (5.2) | 97 (5.6) | 0.02 |
| NSAIDs | 2623 (74.8) | 1315 (75.6) | 0.02 | 2604 (74.7) | 1330 (76.9) | 0.05 | 2640 (75.8) | 1323 (76.5) | 0.02 |
| Proton pump inhibitors | 1001 (28.5) | 514 (29.6) | 0.02 | 996 (28.6) | 530 (30.6) | 0.05 | 1003 (28.8) | 523 (30.2) | 0.03 |
| Renin system inhibitors | 2268 (64.7) | 1108 (63.7) | 0.02 | 2250 (64.6) | 1163 (67.2) | 0.06 | 2243 (64.4) | 1144 (66.1) | 0.04 |
| Statins | 1291 (36.8) | 558 (32.1) | 0.10 | 1278 (36.7) | 648 (37.5) | 0.02 | 1209 (34.7) | 618 (35.7) | 0.02 |
| Number of antidepressants¶ |  |  |  |  |  |  |  |  |  |
| Without medications | 1460 (41.6) | 795 (45.7) | 0.08 | 1452 (41.7) | 710 (41.0) | 0.01 | 1511 (43.4) | 737 (42.6) | 0.02 |
| 1 type | 1797 (51.2) | 831 (47.8) | 0.07 | 1783 (51.2) | 893 (51.6) | 0.01 | 1732 (49.7) | 870 (50.3) | 0.01 |
| More than 2 types | 250 (7.1) | 113 (6.5) | 0.03 | 249 (7.1) | 127 (7.4) | 0.01 | 242 (6.9) | 123 (7.1) | 0.01 |

NOACs, non-vitamin K antagonist oral anticoagulants; SMD, standardized mean differences; ATT, average treatment effect among the treated population; ATE, average treatment effect; COPD, chronic obstructive pulmonary disease; NSAIDs, non-steroidal anti-inflammatory drugs.

* All covariates listed in the table were used to calculate the propensity score with fine stratification weighting.

Data are expressed as number (%) unless otherwise indicated.

† Expressed as mean (SD)

§ Calculated by baseline age [>65 years = 1 point; >75 years =2 points], female, congestive heart failure, hypertension, previous stroke/transient ischemic attack/thromboembolism [2 points], vascular disease, diabetes mellitus
¶ Defined by selective serotonin reuptake inhibitors (SSRIs), serotonin norepinephrine reuptake inhibitors (SNRIs) and others (including mirtazapine, bupropion, agomelatine, vortioxetine, moclobemide)

| **Table S8.** Risk of suicide-related outcomes among AF patients with depressive disorders. | | | | | | |
| --- | --- | --- | --- | --- | --- | --- |
| **Study outcomes*** | **Weighted cohort with ATE** | | | **Weighted cohort with ATT** | | |
|  | Events, n | Incidence rate‡ | HR (95% CI) | Events, n | Incidence rate‡ | HR (95% CI) |
| **Primary outcome** |  |  |  |  |  |  |
| Suicide-related outcomes | |  |  |  |  |  |
| NOACs | 85 | 8.07 | 0.85 (0.49-1.45) | 81 | 6.72 | 0.75 (0.49-1.15) |
| warfarin | 50 | 9.43 | 1.00 (reference) | 54 | 8.94 | 1.00 (reference) |
| **Secondary outcomes** |  |  |  |  |  |  |
| Attempted suicides |  |  |  |  |  |  |
| NOACs | 72 | 6.83 | 1.00 (0.58-1.72) | 68 | 5.64 | 0.81 (0.52-1.25) |
| warfarin | 36 | 6.79 | 1.00 (reference) | 42 | 6.95 | 1.00 (reference) |
| Successful suicide |  |  |  |  |  |  |
| NOACs | 14 | 1.31 | 0.48 (0.13-1.80) | 15 | 1.23 | 0.55 (0.18-1.67) |
| warfarin | 14 | 2.60 | 1.00 (reference) | 13 | 2.12 | 1.00 (reference) |

ATE, average treatment effect; ATT, average treatment effect among the treated population.

* In each outcome analysis, patients who had already experienced the corresponding outcome event before index date were excluded.

‡ Per 1000 person-years

| **Table S9.** Baseline characteristics of the original population and the weighted population excluding AF patients with chronic kidney disease. | | | | | | | | | |
| --- | --- | --- | --- | --- | --- | --- | --- | --- | --- |
| **Baseline characteristics*** | **Original cohort** | | | **Weighted cohort with ATE** | | | **Weighted cohort with ATT** | | |
|  | **NOACs**  **(n=92,828)** | **Warfarin**  **(n=35,998)** | **SMD** | **NOACs**  **(n=92,763)** | **Warfarin**  **(n=35,981)** | **SMD** | **NOACs**  **(n=92,763)** | **Warfarin**  **(n=35,981)** | **SMD** |
| Age, years† | 74 (11.0) | 70 (12.8) | 0.34 | 72.9 (11.4) | 73 (12.4) | 0.01 | 74 (11.0) | 74.2 (12.1) | 0.01 |
| Sex |  |  |  |  |  |  |  |  |  |
| Male | 52065 (56.1) | 21112 (58.6) | 0.05 | 52802 (56.9) | 20455 (56.8) | 0.00 | 52025 (56.1) | 20206 (56.2) | 0.00 |
| Female | 40763 (43.9) | 14886 (41.4) | 0.05 | 39961 (43.1) | 15526 (43.2) | 0.00 | 40738 (43.9) | 15775 (43.8) | 0.00 |
| Insurance premium level |  |  |  |  |  |  |  |  |  |
| <28,800 NTD | 68144 (73.4) | 25810 (71.7) | 0.04 | 67667 (72.9) | 26247 (72.9) | 0.00 | 68084 (73.4) | 26420 (73.4) | 0.00 |
| 28,801-45,800 NTD | 11934 (12.9) | 5653 (15.7) | 0.08 | 12685 (13.7) | 5010 (13.9) | 0.01 | 11933 (12.9) | 4763 (13.2) | 0.01 |
| >45,800 NTD | 12750 (13.7) | 4535 (12.6) | 0.03 | 12411 (13.4) | 4724 (13.1) | 0.01 | 12746 (13.7) | 4798 (13.3) | 0.01 |
| CHA2DS2-VASc score†§ | 3.7 (1.7) | 3.2 (2.0) | 0.26 | 3.6 (1.7) | 3.6 (2.0) | 0.00 | 3.7 (1.7) | 3.7 (2) | 0.01 |
| Index year |  |  |  |  |  |  |  |  |  |
| 2012 to 2014 | 13184 (14.2) | 20347 (56.5) | 0.99 | 24007 (25.9) | 9454 (26.3) | 0.01 | 13183 (14.2) | 5235 (14.5) | 0.01 |
| 2015 to 2017 | 36140 (38.9) | 10777 (29.9) | 0.19 | 34040 (36.7) | 13138 (36.5) | 0.00 | 36140 (39.0) | 14054 (39.1) | 0.00 |
| 2018 to 2020 | 43504 (46.9) | 4874 (13.5) | 0.78 | 34717 (37.4) | 13389 (37.2) | 0.00 | 43440 (46.8) | 16691 (46.4) | 0.01 |
| Hospital level |  |  |  |  |  |  |  |  |  |
| Medical centers | 37241 (40.1) | 12090 (33.6) | 0.14 | 35386 (38.1) | 13096 (36.4) | 0.04 | 37181 (40.1) | 13488 (37.5) | 0.05 |
| Regional hospitals | 39921 (43.0) | 16317 (45.3) | 0.05 | 40663 (43.8) | 16519 (45.9) | 0.04 | 39917 (43.0) | 16600 (46.1) | 0.06 |
| District hospitals or  clinics | 15666 (16.9) | 7591 (21.1) | 0.11 | 16714 (18.0) | 6365 (17.7) | 0.01 | 15665 (16.9) | 5894 (16.4) | 0.01 |
| Specialty of prescriber |  |  |  |  |  |  |  |  |  |
| Cardiology | 66550 (71.7) | 24179 (67.2) | 0.10 | 65279 (70.4) | 25350 (70.5) | 0.00 | 66499 (71.7) | 25806 (71.7) | 0.00 |
| Neurology | 14344 (15.5) | 5130 (14.3) | 0.03 | 14070 (15.2) | 5335 (14.8) | 0.01 | 14330 (15.4) | 5414 (15.0) | 0.01 |
| Others | 11934 (12.9) | 6689 (18.6) | 0.16 | 13414 (14.5) | 5296 (14.7) | 0.01 | 11934 (12.9) | 4761 (13.2) | 0.01 |
| Major bleeding events | 22832 (24.6) | 5992 (16.6) | 0.20 | 20677 (22.3) | 8145 (22.6) | 0.01 | 22802 (24.6) | 8981 (25) | 0.01 |
| Psychiatric comorbidities |  |  |  |  |  |  |  |  |  |
| Anxiety disorders | 9740 (10.5) | 4325 (12) | 0.05 | 10157 (10.9) | 3863 (10.7) | 0.01 | 9738 (10.5) | 3685 (10.2) | 0.01 |
| Bipolar disorders | 254 (0.3) | 105 (0.3) | 0.00 | 258 (0.3) | 109 (0.3) | 0.00 | 254 (0.3) | 110 (0.3) | 0.01 |
| Dementia | 6427 (6.9) | 1985 (5.5) | 0.06 | 6025 (6.5) | 2268 (6.3) | 0.01 | 6416 (6.9) | 2377 (6.6) | 0.01 |
| Schizophrenia | 417 (0.4) | 190 (0.5) | 0.01 | 434 (0.5) | 168 (0.5) | 0.00 | 416 (0.4) | 160 (0.4) | 0.00 |
| Physical comorbidities |  |  |  |  |  |  |  |  |  |
| Anemia | 5718 (6.2) | 1898 (5.3) | 0.04 | 5499 (5.9) | 2093 (5.8) | 0.01 | 5718 (6.2) | 2169 (6) | 0.01 |
| Cataract | 18769 (20.2) | 6476 (18) | 0.06 | 18204 (19.6) | 7101 (19.7) | 0.00 | 18750 (20.2) | 7343 (20.4) | 0.01 |
| COPD | 5050 (5.4) | 4561 (12.7) | 0.25 | 7066 (7.6) | 2795 (7.8) | 0.01 | 5050 (5.4) | 2112 (5.9) | 0.03 |
| Congestive heart failure | 28125 (30.3) | 12342 (34.3) | 0.09 | 29259 (31.5) | 11599 (32.2) | 0.02 | 28109 (30.3) | 11312 (31.4) | 0.00 |
| Coronary artery disease | 33095 (35.7) | 13960 (38.8) | 0.07 | 33957 (36.6) | 13125 (36.5) | 0.00 | 33078 (35.7) | 12805 (35.6) | 0.01 |
| Deep vein thrombosis | 220 (0.2) | 200 (0.6) | 0.05 | 312 (0.3) | 129 (0.4) | 0.00 | 220 (0.2) | 102 (0.3) | 0.01 |
| Diabetes mellitus | 27376 (29.5) | 10187 (28.3) | 0.03 | 27085 (29.2) | 10581 (29.4) | 0.01 | 27362 (29.5) | 10734 (29.8) | 0.01 |
| Epilepsy | 1285 (1.4) | 491 (1.4) | 0.00 | 1280 (1.4) | 477 (1.3) | 0.01 | 1281 (1.4) | 471 (1.3) | 0.01 |
| Glaucoma | 5215 (5.6) | 1626 (4.5) | 0.05 | 4923 (5.3) | 1867 (5.2) | 0.01 | 5207 (5.6) | 1961 (5.5) | 0.03 |
| Hypertension | 56088 (60.4) | 15005 (41.7) | 0.38 | 50803 (54.8) | 19461 (54.1) | 0.01 | 56030 (60.4) | 21190 (58.9) | 0.00 |
| Hyperthyroidism | 3048 (3.3) | 1596 (4.4) | 0.06 | 3355 (3.6) | 1310 (3.6) | 0.00 | 3048 (3.3) | 1201 (3.3) | 0.01 |
| Hypothyroidism | 1815 (2.0) | 669 (1.9) | 0.01 | 1800 (1.9) | 716 (2) | 0.00 | 1813 (2.0) | 734 (2.0) | 0.02 |
| Hyperlipidemia | 32337 (34.8) | 11182 (31.1) | 0.08 | 31359 (33.8) | 11948 (33.2) | 0.01 | 32306 (34.8) | 12246 (34) | 0.01 |
| Malignancy | 15014 (16.2) | 5598 (15.6) | 0.02 | 14747 (15.9) | 5670 (15.8) | 0.00 | 15004 (16.2) | 5699 (15.8) | 0.01 |
| Osteoporosis | 6354 (6.8) | 2136 (5.9) | 0.04 | 6135 (6.6) | 2413 (6.7) | 0.00 | 6339 (6.8) | 2520 (7) | 0.00 |
| Parkinsonism | 2529 (2.7) | 825 (2.3) | 0.03 | 2399 (2.6) | 940 (2.6) | 0.00 | 2524 (2.7) | 985 (2.7) | 0.01 |
| Pulmonary embolism | 484 (0.5) | 482 (1.3) | 0.09 | 646 (0.7) | 260 (0.7) | 0.00 | 484 (0.5) | 176 (0.5) | 0.01 |
| Rheumatoid arthritis | 1092 (1.2) | 449 (1.2) | 0.01 | 1129 (1.2) | 413 (1.1) | 0.01 | 1092 (1.2) | 399 (1.1) | 0.02 |
| Ischemic stroke | 19128 (20.6) | 7720 (21.4) | 0.02 | 19332 (20.8) | 7326 (20.4) | 0.01 | 19116 (20.6) | 7174 (19.9) | 0.01 |
| Medication use |  |  |  |  |  |  |  |  |  |
| Antacids | 21695 (23.4) | 12069 (33.5) | 0.23 | 24243 (26.1) | 9288 (25.8) | 0.01 | 21692 (23.4) | 8212 (22.8) | 0.01 |
| Antiarrthymic drugs | 42876 (46.2) | 18306 (50.9) | 0.09 | 43966 (47.4) | 16808 (46.7) | 0.01 | 42871 (46.2) | 16232 (45.1) | 0.02 |
| Antidepressants | 3641 (3.9) | 1089 (3.0) | 0.05 | 3390 (3.7) | 1302 (3.6) | 0.02 | 3629 (3.9) | 1385 (3.8) | 0.00 |
| Antiepileptics | 10221 (11.0) | 3489 (9.7) | 0.0 | 9862 (10.6) | 3912 (10.9) | 0.01 | 10201 (11.0) | 4076 (11.3) | 0.01 |
| Antiosteoporotic drugs | 7579 (8.2) | 3130 (8.7) | 0.02 | 7643 (8.2) | 2973 (8.3) | 0.00 | 7576 (8.2) | 2914 (8.1) | 0.00 |
| Antiplatelet drugs | 62383 (67.2) | 24539 (68.2) | 0.02 | 62860 (67.8) | 24759 (68.8) | 0.02 | 62322 (67.2) | 24846 (69.1) | 0.04 |
| Antipsychotics | 9042 (9.7) | 3381 (9.4) | 0.01 | 8916 (9.6) | 3429 (9.5) | 0.00 | 9035 (9.7) | 3449 (9.6) | 0.01 |
| Anxiolytics | 37914 (40.8) | 15498 (43.1) | 0.05 | 38437 (41.4) | 14915 (41.5) | 0.00 | 37889 (40.8) | 14692 (40.8) | 0.00 |
| Beta blockers | 61213 (65.9) | 23815 (66.2) | 0.01 | 61230 (66.0) | 23736 (66.0) | 0.01 | 61177 (65.9) | 23709 (65.9) | 0.00 |
| Bronchodilators | 13009 (14) | 4760 (13.2) | 0.02 | 12832 (13.8) | 5146 (14.3) | 0.01 | 12989 (14.0) | 5296 (14.7) | 0.02 |
| Calcium-channel  blockers | 54295 (58.5) | 21292 (59.1) | 0.01 | 54356 (58.6) | 21181 (58.9) | 0.01 | 54247 (58.5) | 21139 (58.8) | 0.01 |
| Corticosteroids | 25374 (27.3) | 10103 (28.1) | 0.02 | 25619 (27.6) | 9961 (27.7) | 0.00 | 25353 (27.3) | 9908 (27.5) | 0.01 |
| Diuretics | 41098 (44.3) | 17726 (49.2) | 0.10 | 42401 (45.7) | 16749 (46.6) | 0.02 | 41071 (44.3) | 16373 (45.5) | 0.03 |
| H2 blockers | 44067 (47.5) | 16081 (44.7) | 0.06 | 43352 (46.7) | 17032 (47.3) | 0.01 | 44030 (47.5) | 17403 (48.4) | 0.02 |
| Hypnotics and sedatives | 18933 (20.4) | 7915 (22.0) | 0.04 | 19299 (20.8) | 7534 (20.9) | 0.00 | 18917 (20.4) | 7388 (20.5) | 0.00 |
| Hypoglycemic agents | 25556 (27.5) | 9500 (26.4) | 0.03 | 25256 (27.2) | 9847 (27.4) | 0.00 | 25541 (27.5) | 9981 (27.7) | 0.01 |
| Mood stabilizers | 1719 (1.9) | 710 (2.0) | 0.01 | 1750 (1.9) | 679 (1.9) | 0.00 | 1716 (1.8) | 668 (1.9) | 0.00 |
| NSAIDs | 61330 (66.1) | 24612 (68.4) | 0.05 | 61813 (66.6) | 24015 (66.7) | 0.00 | 61293 (66.1) | 23789 (66.1) | 0.00 |
| Proton pump inhibitors | 18612 (20.0) | 6752 (18.8) | 0.03 | 18292 (19.7) | 7146 (19.9) | 0.00 | 18597 (20.0) | 7299 (20.3) | 0.01 |
| Renin system inhibitors | 58227 (62.7) | 21831 (60.6) | 0.04 | 57639 (62.1) | 22344 (62.1) | 0.00 | 58169 (62.7) | 22544 (62.7) | 0.00 |
| Statins | 32929 (35.5) | 10259 (28.5) | 0.15 | 31128 (33.6) | 11984 (33.3) | 0.01 | 32892 (35.5) | 12654 (35.2) | 0.01 |

NOACs, non-vitamin K antagonist oral anticoagulants; SMD, standardized mean differences; ATE, average treatment effect; ATT, average treatment effect among the treated population; COPD, chronic obstructive pulmonary disease; NSAIDs, non-steroidal anti-inflammatory drugs.

* All covariates listed in the table were used to calculate the propensity score with fine stratification weighting.

Data are expressed as number (%) unless otherwise indicated.

† Expressed as mean (SD)

§ Calculated by baseline age [>65 years = 1 point; >75 years =2 points], female, congestive heart failure, hypertension, previous stroke/transient ischemic attack/thromboembolism [2 points], vascular disease, diabetes mellitus

| **Table S10.** Risk of suicide-related outcomes excluding AF patients with chronic kidney disease. | | | | | | |
| --- | --- | --- | --- | --- | --- | --- |
| **Study outcomes*** | **Weighted cohort with ATE** | | | **Weighted cohort with ATT** | | |
|  | Events, n | Incidence rate‡ | HR (95% CI) | Events, n | Incidence rate‡ | HR (95% CI) |
| **Primary outcome** |  |  |  |  |  |  |
| Suicide-related outcomes | |  |  |  |  |  |
| NOACs | 1,169 | 3.89 | 0.86 (0.74-1.00) | 1,096 | 4.22 | 0.81 (0.68-0.97) |
| warfarin | 537 | 4.54 | 1.00 (reference) | 534 | 5.23 | 1.00 (reference) |
| **Secondary outcomes** |  |  |  |  |  |  |
| Attempted suicides |  |  |  |  |  |  |
| NOACs | 1,055 | 3.51 | 0.83 (0.71-0.97) | 997 | 3.84 | 0.78 (0.65-0.94) |
| warfarin | 500 | 4.23 | 1.00 (reference) | 503 | 4.92 | 1.00 (reference) |
| Successful suicide |  |  |  |  |  |  |
| NOACs | 126 | 0.42 | 1.21 (0.75-1.97) | 109 | 0.42 | 1.25 (0.65-2.40) |
| warfarin | 40 | 0.34 | 1.00 (reference) | 34 | 0.33 | 1.00 (reference) |

ATE, average treatment effect; ATT, average treatment effect among the treated population.

* In each outcome analysis, patients who had already experienced the corresponding outcome event before index date were excluded.

‡ Per 1000 person-years

| **Table S11.** Risk of suicide-related outcomes applying on treatment analysis. | | | | | | |
| --- | --- | --- | --- | --- | --- | --- |
| **Study outcomes*** | **Weighted cohort with ATE** | | | **Weighted cohort with ATT** | | |
|  | Events, n | Incidence rate‡ | HR (95% CI) | Events, n | Incidence rate‡ | HR (95% CI) |
| **Primary outcome** |  |  |  |  |  |  |
| Suicide-related outcomes | |  |  |  |  |  |
| NOACs | 770 | 4.11 | 0.79 (0.60-1.05) | 771 | 4.47 | 0.73 (0.53-1.02) |
| warfarin | 162 | 5.49 | 1.00 (reference) | 174 | 6.56 | 1.00 (reference) |
| **Secondary outcomes** |  |  |  |  |  |  |
| Attempted suicides |  |  |  |  |  |  |
| NOACs | 704 | 3.76 | 0.77 (0.58-1.03) | 712 | 4.13 | 0.71 (0.51-1.00) |
| warfarin | 153 | 5.18 | 1.00 (reference) | 166 | 6.26 | 1.00 (reference) |
| Successful suicide |  |  |  |  |  |  |
| NOACs | 73 | 0.39 | 1.32 (0.52-3.33) | 64 | 0.37 | 1.33 (0.38-4.63) |
| warfarin | 9 | 0.30 | 1.00 (reference) | 8 | 0.30 | 1.00 (reference) |

ATE, average treatment effect; ATT, average treatment effect among the treated population.

* In each outcome analysis, patients who had already experienced the corresponding outcome event before index date were excluded.

‡ Per 1000 person-years

| **Table S12.** Risk of suicide-related outcomes using subdistribution hazards models. | | |
| --- | --- | --- |
| **Study outcomes*** | **Weighted cohort with ATE** | **Weighted cohort with ATT** |
|  | sdHR (95% CI) | sdHR (95% CI) |
| **Primary outcome** |  |  |
| Suicide-related outcomes | |  |
| NOACs | 0.89 (0.81-0.98) | 0.86 (0.78-0.94) |
| warfarin | 1.00 (reference) | 1.00 (reference) |
| **Secondary outcomes** |  |  |
| Attempted suicides |  |  |
| NOACs | 0.87 (0.79-0.96) | 0.83 (0.75-0.91) |
| warfarin | 1.00 (reference) | 1.00 (reference) |
| Successful suicide |  |  |
| NOACs | 1.23 (0.89-1.70) | 1.34 (0.94-1.91) |
| warfarin | 1.00 (reference) | 1.00 (reference) |

ATE, average treatment effect; ATT, average treatment effect among the treated population; sdHR, subdistribution hazard ratio.

* In each outcome analysis, patients who had already experienced the corresponding outcome event before index date were excluded.

‡ Per 1000 person-years

| **Table S13.** Risk of positive control outcomes and negative control outcomes. | | | | | | |
| --- | --- | --- | --- | --- | --- | --- |
| **Study outcomes*** | **Weighted cohort with ATT** | | | **Weighted cohort with ATE** | | |
|  | Events, n | Incidence rate‡ | HR (95% CI) | Events, n | Incidence rate‡ | HR (95% CI) |
| **Positive control outcomes** | |  |  |  |  |  |
| Ischemic stroke/systemic embolism | | |  |  |  |  |
| NOACs | 9,285 | 38.64 | 0.83 (0.79-0.88) | 8,306 | 39.57 | 0.81 (0.76-0.86) |
| warfarin | 4,258 | 46.17 | 1.00 (reference) | 3,944 | 48.90 | 1.00 (reference) |
| All-cause mortality |  |  |  |  |  |  |
| NOACs | 27,363 | 82.61 | 0.78 (0.76-0.80) | 24,954 | 87.40 | 0.76 (0.74-0.79) |
| warfarin | 13,907 | 105.91 | 1.00 (reference) | 13,023 | 114.52 | 1.00 (reference) |
| Cardiovascular mortality | |  |  |  |  |  |
| NOACs | 10,853 | 32.77 | 0.72 (0.69-0.75) | 9,899 | 34.67 | 0.70 (0.66-0.73) |
| warfarin | 5,963 | 45.41 | 1.00 (reference) | 5,644 | 49.63 | 1.00 (reference) |
| **Negative control outcomes** | |  |  |  |  |  |
| Oral health-related conditions | | |  |  |  |  |
| NOACs | 12,445 | 44.87 | 1.01 (0.97-1.06) | 10,904 | 45.02 | 1.00 (0.95-1.05) |
| warfarin | 4,854 | 44.05 | 1.00 (reference) | 4,331 | 44.85 | 1.00 (reference) |
| Suicide-related outcomes within 90 days | | |  |  |  |  |
| NOACs | 148 | 6.03 | 1.52 (0.92-2.51) | 146 | 5.98 | 1.66 (0.86-3.20) |
| warfarin | 38 | 3.96 | 1.00 (reference) | 34 | 3.56 | 1.00 (reference) |

ATE, average treatment effect; ATT, average treatment effect among the treated population.

* In each outcome analysis, patients who had already experienced the corresponding outcome event before index date were excluded.

‡ Per 1000 person-years

| **Fig S1.** Study design diagram. |
| --- |
| **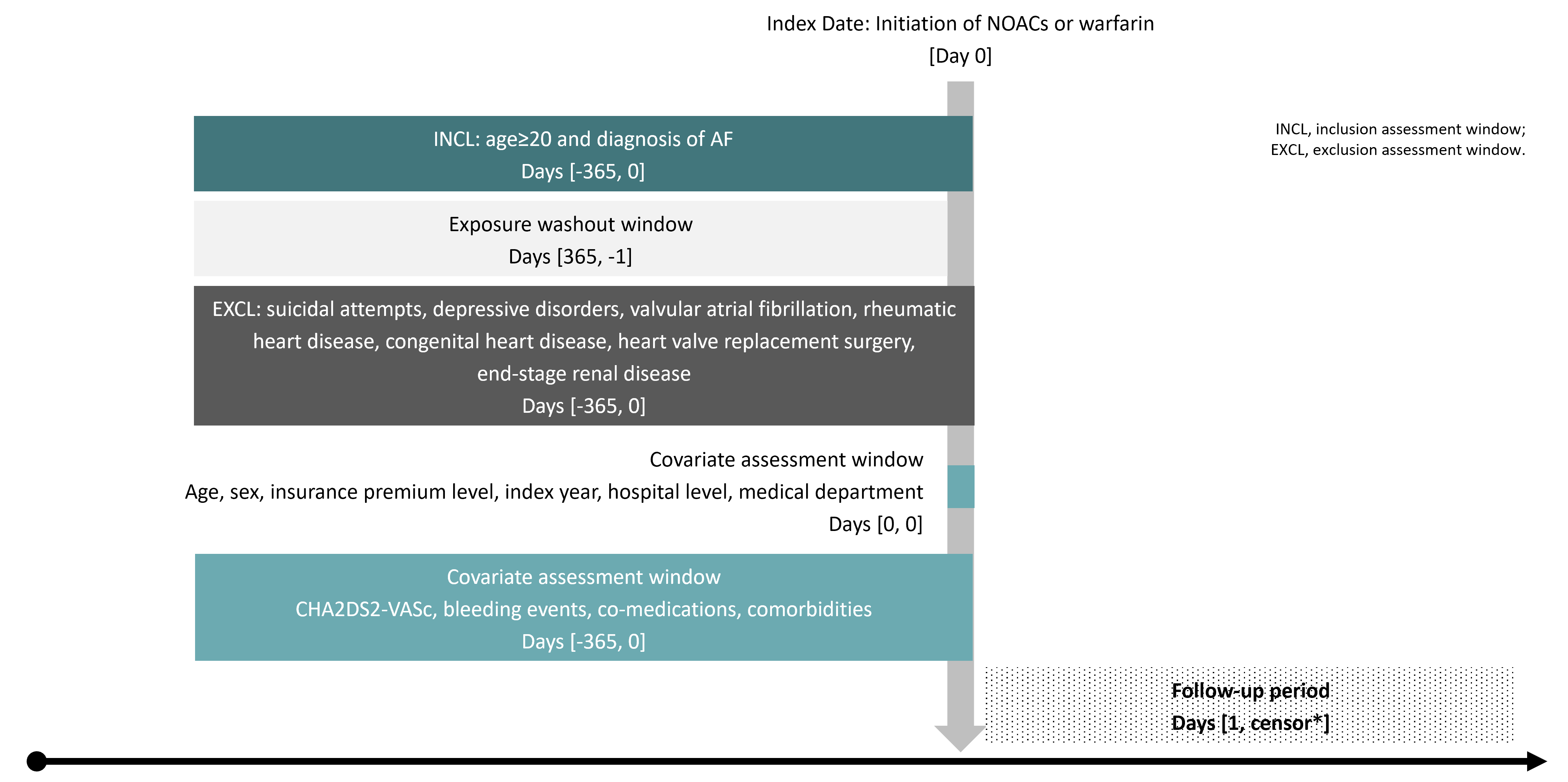** |
| INCL, inclusion assessment window; EXCL, exclusion assessment window.  * Censoring events were defined as the occurrence of study outcomes, at loss to follow up, at death, or the end of the study period (December 31, 2020), whichever occurs first. |

| **Fig S2.** Distribution of propensity score before and after propensity score weighting. |
| --- |
| 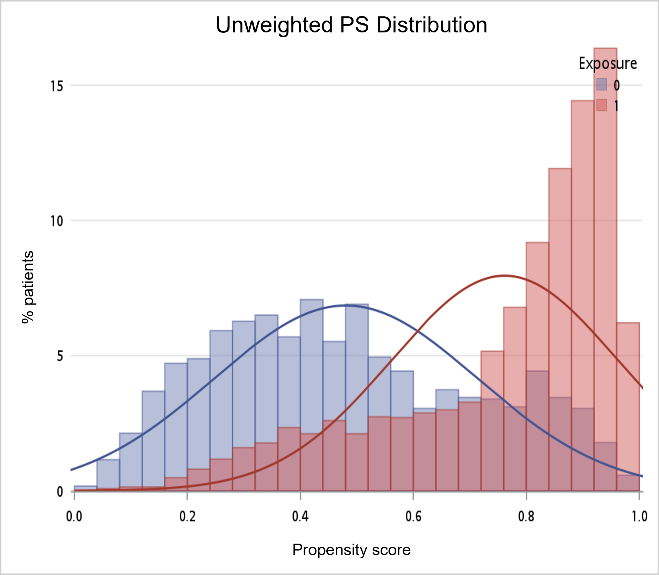 |
| 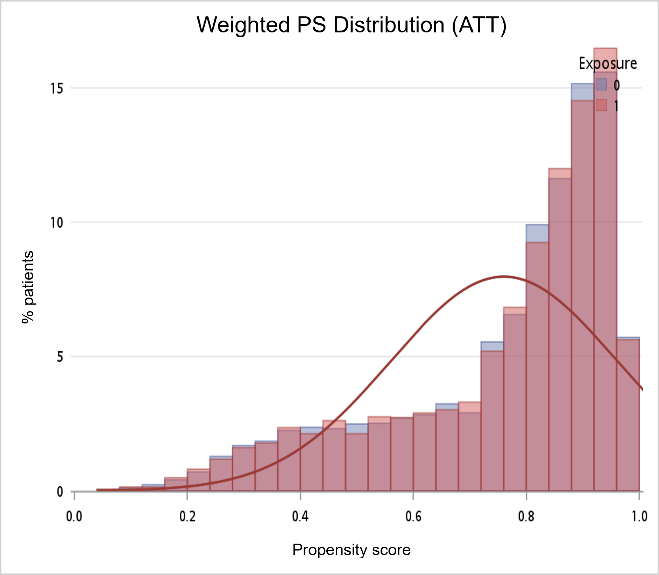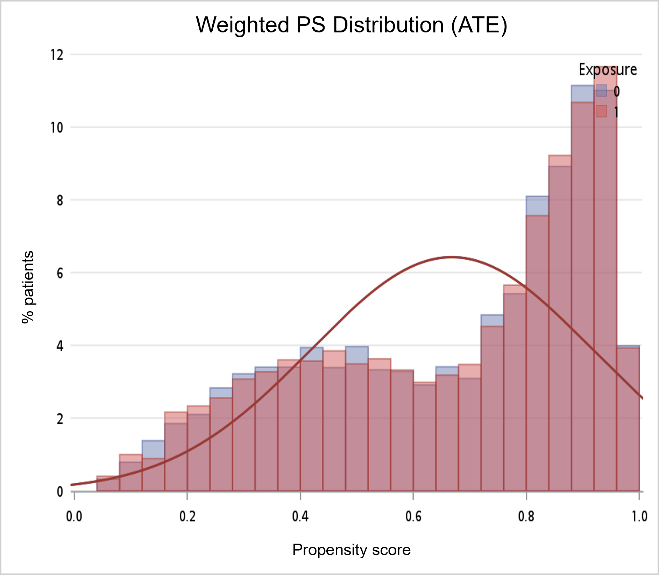 |
| ATT, average treatment effect among the treated population; ATE, average treatment effect.  * Red bar indicates the PS distribution of NOACs users; blue bar indicates the PS distribution of warfarin users. |

| **Fig S3.** Covariate balance measured by standardized mean differences before and after propensity score weighting. |
| --- |
| 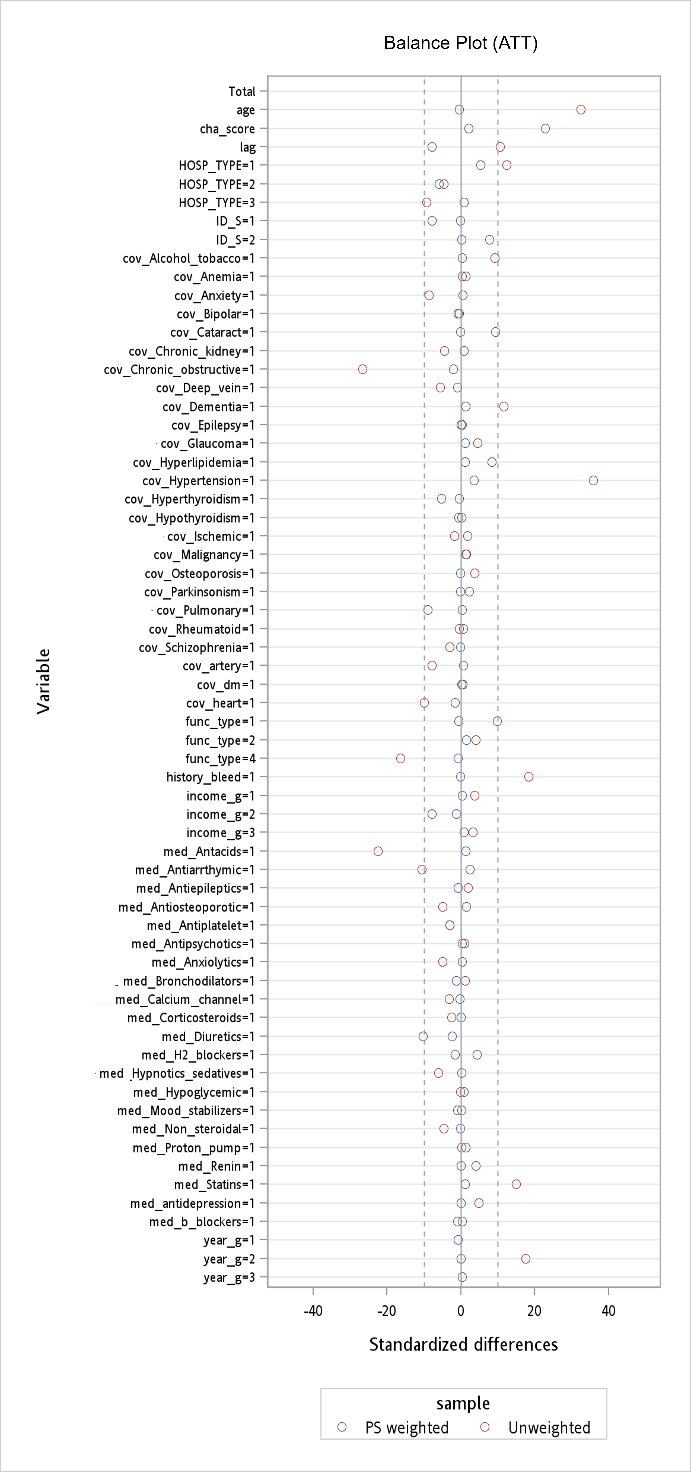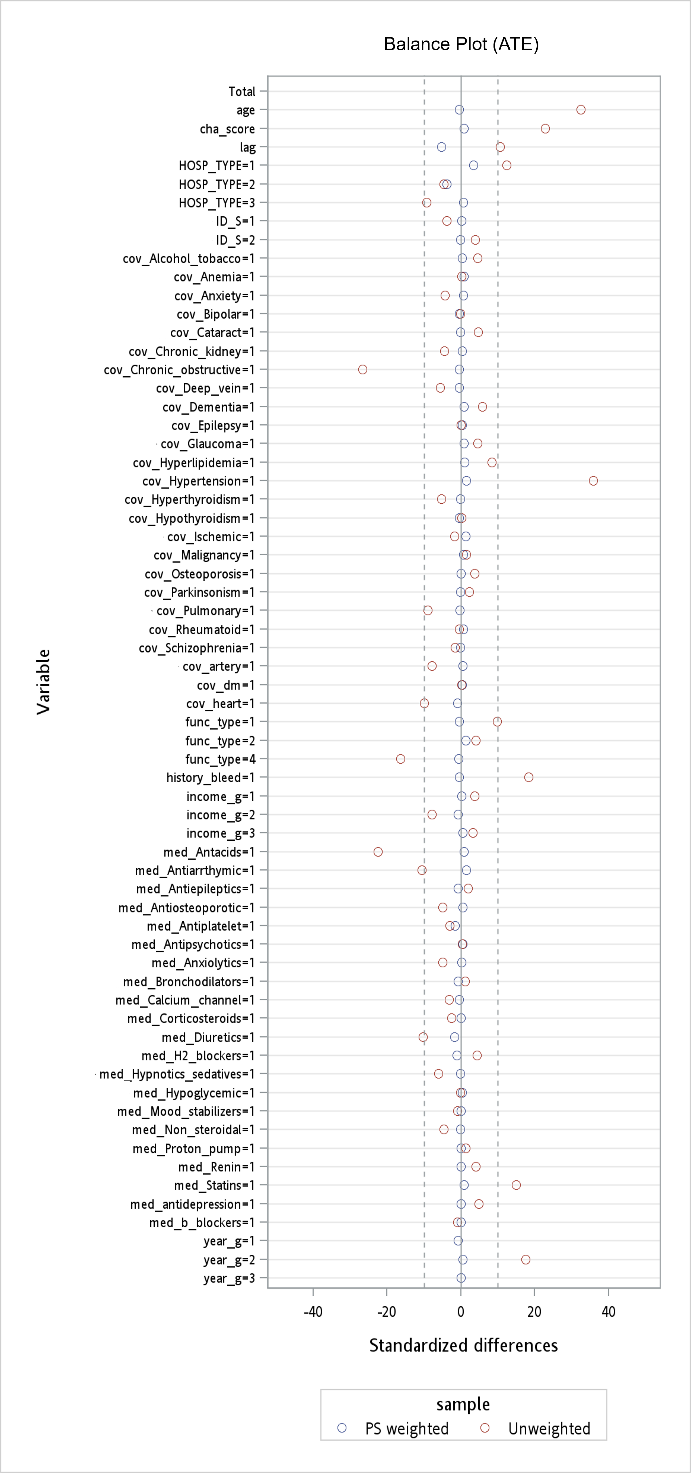 |
| ATT, average treatment effect among the treated population; ATE, average treatment effect; cha_score, CHA2DS2-VASc score; lag, time from AF diagnosis to OAC prescription; hospital_type, hospital level; ID_S, sex; func_type, medical department; income_g, insurance premium level; year_g, index year. |
